# Identification of target antigens of anti-endothelial cell and anti-vascular smooth muscle cell antibodies in patients with giant cell arteritis: a proteomic approach

**DOI:** 10.1186/ar3388

**Published:** 2011-06-28

**Authors:** Alexis Régent, Hanadi Dib, Kim H Ly, Christian Agard, Mathieu C Tamby, Nicolas Tamas, Babette Weksler, Christian Federici, Cédric Broussard, Loïc Guillevin, Luc Mouthon

**Affiliations:** 1 Inserm U1016, Institut Cochin, CNRS UMR 8104, 8 rue Méchain, F-75014 Paris, France; 2 Université Paris Descartes, 12 rue de l'Ecole de Médecine, F-75270 Paris, France; 3 Pôle de Médecine Interne, Centre de Référence pour les vascularites nécrosantes et la sclérodermie systémique, Hôpital Cochin, Assistance Publique Hôpitaux de Paris, 27 rue du Faubourg Saint-Jacques, F-75679 Paris Cedex 14 Paris, France; 4 Service de Médecine Interne A, CHU Dupuytren, 2 avenue Martin Luther King, F-87042 Limoges cedex 1, France; 5 Service de Médecine Interne, hôpital Hôtel Dieu, Place Alexis Ricordeau, F-44093 Nantes cedex 1, France; 6 Weill Medical College of Cornell University, 1300 York Avenue, New York, NY 10065, USA; 7 Institut Cochin, Plate-forme Protéomique de l'Université Paris Descartes, CNRS UMR 8104, 8 rue Méchain, F-75014 Paris, France

## Abstract

**Introduction:**

Immunological studies of giant cell arteritis (GCA) suggest that a triggering antigen of unknown nature could generate a specific immune response. We thus decided to detect autoantibodies directed against endothelial cells (ECs) and vascular smooth muscle cells (VSMCs) in the serum of GCA patients and to identify their target antigens.

**Methods:**

Sera from 15 GCA patients were tested in 5 pools of 3 patients' sera and compared to a sera pool from 12 healthy controls (HCs). Serum immunoglobulin G (IgG) reactivity was analysed by 2-D electrophoresis and immunoblotting with antigens from human umbilical vein ECs (HUVECs) and mammary artery VSMCs. Target antigens were identified by mass spectrometry.

**Results:**

Serum IgG from GCA patients recognised 162 ± 3 (mean ± SD) and 100 ± 17 (mean ± SD) protein spots from HUVECs and VSMCs, respectively, and that from HCs recognised 79 and 94 protein spots, respectively. In total, 30 spots from HUVECs and 19 from VSMCs were recognised by at least two-thirds and three-fifths, respectively, of the pools of sera from GCA patients and not by sera from HCs. Among identified proteins, we found vinculin, lamin A/C, voltage-dependent anion-selective channel protein 2, annexin V and other proteins involved in cell energy metabolism and key cellular pathways. Ingenuity pathway analysis revealed that most identified target antigens interacted with growth factor receptor-bound protein 2.

**Conclusions:**

IgG antibodies to proteins in the proteome of ECs and VSMCs are present in the sera of GCA patients and recognise cellular targets that play key roles in cell biology and maintenance of homeostasis. Their potential pathogenic role remains to be determined.

## Introduction

Giant cell arteritis (GCA), also known as temporal arteritis, is a primary systemic vasculitis involving large- and medium-sized vessels. GCA commonly causes bitemporal headaches, jaw claudication, scalp tenderness and/or abnormal temporal arteries (tender, nodular, swollen and thickened arteries with decreased pulses) detected during physical examinations. GCA does not occur in people younger than 50 years old, and its incidence increases with age and peaks in Caucasians older than 70 years of age [1,2]. Ocular ischaemic complications occur in 25% of the patients and leads to irreversible visual loss in 15% [3]. No definite immunological marker has been identified in GCA, and patients usually present with increased erythrocyte sedimentation rates and/or C-reactive protein levels. Diagnosing GCA can be difficult, and temporal artery biopsy is the gold standard for making the diagnosis [4]. However, in 10% to 20% of patients with GCA, the biopsy shows no specific change [5].

GCA is an inflammatory condition of unknown origin characterised by the presence of giant cells and a remodelling process in the arterial wall [6]. In patients with GCA, an immune-mediated reaction is suspected to be triggered by an antigen of unknown origin, either microbial or a self-antigen, that could be presented to T cells by dendritic cells [7]. Thus, macrophages and giant cells stimulated by interferon-γ (IFN-γ) play a major role in the disruption of the elastic lamina and the remodelling of vessel walls. In addition, in the adventitia, macrophages produce proinflammatory cytokines such as interleukin 1 (IL-1) and IL-6, whereas in the media and intima they contribute to arterial injury by producing metalloproteinases and nitric oxide [6,8,9].

Anti-endothelial cell (anti-EC) antibodies (AECAs) have been detected in a wide range of systemic inflammatory and/or autoimmune diseases, including primary and/or secondary systemic vasculitis [10]. Although the pathogenic role of AECAs remains controversial [11,12], these antibodies may be responsible for EC activation [13] and induction of antibody-dependent, cell-mediated cytotoxicity and apoptosis [14]. In GCA, AECAs were detected in 33% of sera by performing ELISA on fixed human umbilical vein ECs (HUVECs) [15], but their presence was not confirmed by indirect immunofluorescence [16]. Anti-vascular smooth muscle cell (anti-VSMC) antibodies have been detected in an experimental rat model of vasculitis [17]; however, to our knowledge, these antibodies have not been investigated in patients with primary systemic vasculitis.

We used 1-D and 2-D immunoblotting, followed by mass spectrometry (MS), to investigate the presence of autoantibodies directed against ECs and VSMCs and identify their target antigens in patients with GCA.

## Materials and methods

### Patients

Serum samples were obtained from 15 patients who fulfilled the American College of Rheumatology (ACR) criteria for GCA [4] and 33 patients with anti-neutrophil cytoplasm antibody (ANCA)-associated vasculitis who fulfilled the ACR and the Chapel Hill criteria used as vasculitis controls, with the control group comprising 15 patients with Wegener's granulomatosis (WG), 9 with Churg-Strauss syndrome (CSS) and 9 with microscopic polyangiitis (MPA) [18]. In each group of patients with ANCA-associated vasculitis, two-thirds of the patients had active disease as assessed by a Birmingham Vasculitis Activity Score (BVAS) >3 in the absence of treatment, and one-third of the patients had inactive disease as assessed by a BVAS <3. Some patients in both groups either received corticosteroids and/or immunsuppressants at the time of blood sampling. Sera from 12 healthy blood donors were used as healthy controls (HCs). Serum samples were collected from patients and HCs, aliquoted and stored at -80°C until use. Serum samples were used individually for 1-D immunoblotting and pooled for 2-D immunoblotting (five pools of sera from three patients with GCA each, and one pool of sera from twelve HCs). All patients and healthy controls gave their written informed consent to participate in the study. Serum samples were collected with the approval of the ethics committee of the groupe hospitalier Pitié-Salpêtrière, and the study conformed to the principles outlined in the Declaration of Helsinki.

### Cell culture

Human internal mammary artery VSMCs were obtained from patients undergoing aortocoronary bypass surgery. All patients gave their written consent, and the protocol for waste surgical tissue was approved by the ethics committee of groupe hospitalier Pitié-Salpêtrière. These cells were immortalised by transduction of a lentiviral vector incorporating the catalytic subunit of the human holoenzyme telomerase RT and T antigen of simian virus 40 in a primary culture of VSMCs as previously described [19]. Immortalised VSMCs were cultured in Smooth Muscle Cell Basal Medium (PromoCell, Heidelberg, Germany) supplemented with decomplemented FCS (5%), insulin (5 μg/mL), basic fibroblast growth factor (bFGF) (2 ng/mL), epidermal growth factor (EGF) (0.5 ng/ml), streptomycin/penicillin (1%) and ciprofloxacin (1%) at 37°C in 5% CO_2_. The VSMC phenotype was confirmed by using smooth muscle myosin heavy chains 1 and 2 and sm22α antibodies (Abcam, Cambridge, UK) (data not shown).

HUVECs were isolated from sterile, freshly obtained umbilical cords at the time of a normal delivery by using 15 mg/mL collagenase type I digestion as previously described [20,21]. All donors gave their written consent. HUVECs were cultured with EC medium (PromoCell) supplemented with decomplemented FCS (2%), bFGF (1 ng/mL), EGF (0.1 ng/mL), EC growth supplement/heparin (0.4%), hydrocortisone (1 μg/mL), streptomycin/penicillin (1%) and ciprofloxacin (1%) at 37°C in 5% CO_2_. HUVECs from four donors were harvested after the third passage to perform protein extraction.

### One-dimensional immunoblotting

Confluent VSMCs were detached with the use of 0.05% trypsin and 0.53 mM ethylenediaminetetraacetic acid. Protein extract was obtained by use of a 125 mM Tris, pH 6.8, solution containing 4% SDS, 1.45 M β-mercaptoethanol, 1 μg/mL aprotinin, 1 μg/mL leupeptin, 1 μg/mL pepstatin and 1 mM phenylmethylsulphonyl fluoride (PMSF). Protein extract was then sonicated four times for 30 seconds each and boiled. In total, 120 μL of solubilised proteins were separated by electrophoresis on 10% SDS-PAGE gels (Bio-Rad Laboratories, Hercules, CA, USA), transferred onto nitrocellulose membranes by using a semidry electroblotter (model A; Ancos, Hojby, Denmark) and incubated with sera from patients with GCA, WG, CSS and MPA or from healthy donors at a 1:100 dilution overnight at 4°C with the Cassette Miniblot System (Immunetics Inc., Cambridge, MA, USA). Detection of IgG reactivity was carried out as previously reported [22-24] (Additional file 1) with the use of a γ-chain-specific secondary rabbit anti-human IgG antibody coupled to alkaline phosphatase. Immunoreactivity was revealed by Nitro Blue Tetrazolium/5-bromo-4-chloro-3-indolyl phosphate staining (Sigma-Aldrich, St. Louis, MO, USA) as previously reported [23,24] (Additional file 1) and quantified by densitometry in reflective mode (Epson Perfection 1200 S densitometer; Seiko Epson Corp., Nagano-ken, Japan) and scanned again to quantify transferred proteins [23,24].

### Two-dimensional immunoblotting

#### Protein extracts

HUVECs and VSMCs were stored at -80°C in 1 mM PMSF and protease inhibitors (Complete Mini; Roche Diagnostics, Meylan, France). Protein extraction was performed as described previously [25] (Additional file 1). Briefly, cells were suspended at 1 × 10^6^/mL in a sample solution extraction kit (Kit 3; Bio-Rad Laboratories). Cell samples were sonicated, and the supernatant was collected after ultracentrifugation (Optima L90-K ultracentrifuge; Beckman Coulter, Fullerton, CA, USA) at 150,000 × *g *for 25 minutes at 4°C. Protein quantification was carried out using the Lowry method [26]. The supernatant was aliquoted and stored at -80°C.

#### Two-dimensional electrophoresis

Two-dimensional electrophoresis (2-DE), 2-D immunoblotting and protein identification by MS were performed as previously reported [27] and are detailed in Additional file 1.

### Modelling with the use of ingenuity pathway analysis software

To gain insight into the biological pathways and networks that were significantly represented in our proteomic data sets, we used ingenuity pathway analysis software (IPA; Ingenuity Systems, Redwood City, CA, USA). IPA selects 'focus proteins' to be used for generating biological networks. Focus proteins are the proteins from data sets that are mapped to corresponding gene objects in the Ingenuity Pathway Knowledgebase (IPKB) and are known to interact with other proteins on the basis of published, peer-reviewed content in the IPKB. From these interactions, IPA builds networks with a size of no more than 35 genes or proteins. A *P *value for each network is calculated according to the fit of the user's set of significant genes and/or proteins. IPA computes a score for each network from the *P *value that indicates the likelihood of the focus proteins in a network being found together by chance. We selected only networks scoring ≥ 2 with *P *< 0.01 of not being generated by chance. Biological functions were assigned to each network by use of annotations from the scientific literature and stored in the IPKB. Fisher's exact test was used to calculate the *P *value to determine the probability of each biological function and/or disease or pathway being assigned by chance. We used *P *≤ 0.05 to select highly significant biological functions and pathways represented in our proteomic data sets. The build function of IPA allows the generation of pathways that can complete the data analysis by showing interactions of identified proteins with a specific group of molecules [28,29].

## Results

The clinical and histological characteristics of patients with GCA are summarised in Additional file 2, Supplemental Table S1. The mean age (± SD) of the patients with GCA was 74.8 ± 8.15 years. Among the 15 patients (5 men), 13 had histological evidence of GCA. All the 15 patients had active disease at the time of blood sampling: twelve were included at the time of diagnosis, two experienced a disease relapse and another one had an acute flare while being treated with prednisone. None of the other 14 patients were taking corticosteroids at the time of blood sampling.

### One-dimensional immunoblotting of IgG reactivity against VSMC protein extracts

One-dimensional immunoblots of IgG reactivity were analysed with VSMC protein extracts in sera from patients with GCA; control patients with ANCA-associated vasculitis, including those with WG, MPA and CSS; and HCs. All subjects tested expressed an IgG reactivity band directed against a 45-kDa protein. In patients with GCA, a number of IgG reactivities were expressed that were not identified in patients with ANCA-associated vasculitis or in HCs, including reactivities directed against protein bands of 85 kDa (Additional file 3).

### Two-dimensional immunoblotting of IgG reactivity against VSMC protein extracts

**Figure 1:**
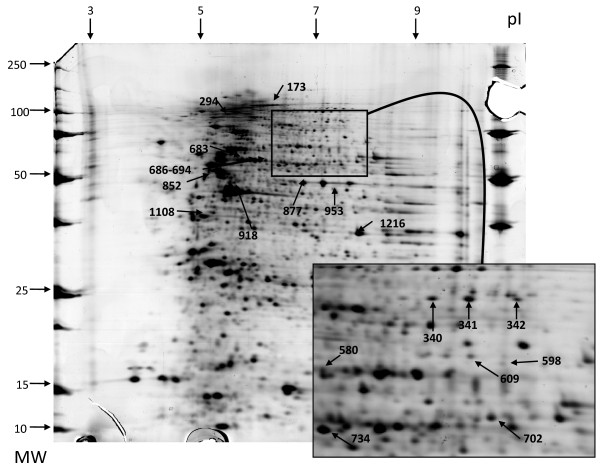
**Two-dimensional silver-stained gel of total protein extracted from vascular smooth muscle cells**. Localisation of the 19 IgG-reactive spots recognised by three-fifths of the pools of sera from giant cell arteritis patients. Numbers were arbitrarily assigned by a computer program. Inset: Enlarged area ranging from 6.5 to 8.2 isoelectric points and 50 to 110 kDa. MW: molecular weight, pI: isoelectric points.

**Figure 2:**
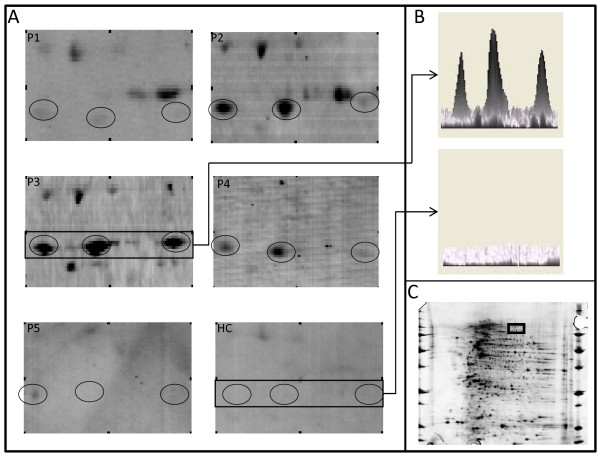
**Serum IgG reactivity to far upstream element-binding protein 2 in sera of giant cell arteritis patients**. Protein extract is from vascular smooth muscle cells (VSMCs). **(A) **IgG reactivity to far upstream element-binding protein 2 (FUBP2) in five different pools of sera from three giant cell arteritis patients each (P1 to P5) and one pool from twelve healthy controls (HC). **(B) **FUBP2 spots are expressed in 3-D view for one representative serum pool of patients (top) and the HC pool (bottom). **(C) **Proteome of VSMCs showing the localisation of FUBP2 spots displayed in **(A)**.

**Figure 3:**
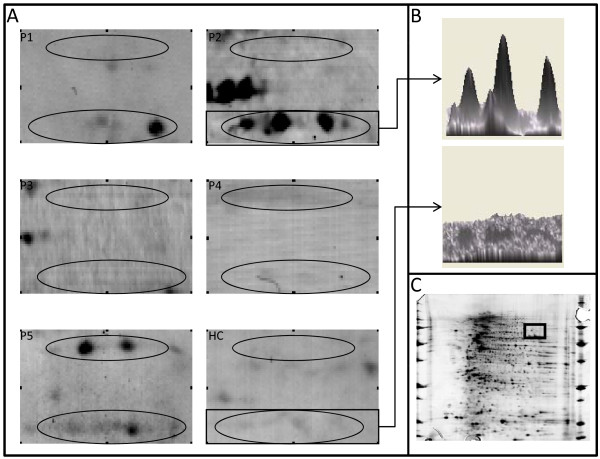
**Serum IgG reactivity to lamin in serum of giant cell arteritis patients**. Protein extract is from vascular smooth muscle cells (VSMCs). **(A) **IgG reactivity to lamin in five different pools of sera from three giant cell arteritis patients each (P1 to P5) and one pool from twelve healthy controls (HC). **(B) **Lamin spots are expressed in 3-D views for one representative sera pool of giant cell arteritis patients (top) and the HC pool (bottom). **(C) **Proteome of VSMCs showing the localisation of lamin spots displayed in **(A)**.

The proteome of VSMCs contained 1,427 different proteins ranging from 3 to 10 isoelectrofocalisation points (IPs) and from 10 to 250 kDa. Among those, a mean (± SD) of 679 ± 258 protein spots were detected after being transferred onto polyvinylidene fluoride (PVDF) membranes. Serum IgG from the HC pool recognised 94 protein spots, whereas IgG from the 5 pools from GCA patients recognised a mean (± SD) of 100 ± 17 protein spots corresponding to a total of 268 different protein spots. Most of these 268 protein spots were recognised from only 1 or 2 pools from patients with GCA and/or from the HC pool. Among these protein spots, 29 were recognised by at least three-fifths of the pools from GCA patients, including 19 not recognised by the HC pool (Additional file 4, Supplemental Table S2). These 19 protein spots were identified by MS as detailed in Table 1 and Additional file 5. The localisations of identified protein spots in the analytical gel are depicted in Figure 1. Among these proteins, only one, the far upstream element-binding protein 2 (FUBP2) (Figure 2), was recognised in all five pools of sera from GCA patients, whereas three different proteins were identified in four pools of sera from GCA patients: actin cytoplasmic 1, actin cytoplasmic 2 and ANKRD26-like family C member 1A (Additional file 4, Supplemental Table S2). Interestingly, IgG from pools of sera from each of three GCA patients recognised lamin A/C (Figure 3) and vinculin (Additional file 6).

**Table 1:** Mass spectrometry data of vascular smooth muscle cell protein spots identified as specific target antigen^a^

Spot ID	Protein	SwissProt accession number	Theoretical/estimated MW, kDa	Theoretical/estimated pI	Number of unique identified peptides^c, d^	Total ion score^d^	Best ion score^d^	Sequence coverage, %^d^
173	Vinculin	[Swiss Prot:VINC_HUMAN]	124/122	5.5/6.4	3/14	39	19	19
294	Putative heat shock protein HSP90, subunit α_2_^b^	[Swiss Prot:HS902_HUMAN]	39/94	4.6/5.6	1/5	40	40	22
340	Far upstream element-binding protein 2	[Swiss Prot:FUBP2_HUMAN]	73/88	6.8/7.2	4-2/10-9	67-46	24-32	21-16
341	Far upstream element-binding protein 2	[Swiss Prot:FUBP2_HUMAN]	73/88	6.8/7.4	5-5/12-11	84-119	24-35	25-19
344	Far upstream element-binding protein 2	[Swiss Prot:FUBP2_HUMAN]	73/88	6.8/7.8	4/11	85	37	22
580	Lamin A/C^b^	[Swiss Prot:LMNA_HUMAN]	74/67	6.6/6.5	1/10	24	24	16
	Coatomer subunit α^b^	[Swiss Prot:COPA_HUMAN]	138/67	7.7/6.5	1/3	37	37	3
598	UDP-glucose 6-dehydrogenase^b^	[Swiss Prot:UGDH_HUMAN]	55/66	6.6/6.7	2/4	46	39	11
609	No identified protein		/66	/5.9				
683	No identified protein		/60	/5.8				
686	Protein disulphide-isomerase A3	[Swiss Prot:PDIA3_HUMAN]	57/59	6.0/6.1	11-11/16-17	1,048-804	140-106	42-44
694	Protein disulphide-isomerase A3	[Swiss Prot:PDIA3_HUMAN]	57/59	6.0/6.3	8-8/14-13	460-362	86-63	38-35
702	No identified protein		/59	/7.6				
734	T-complex protein 1, subunit β	[Swiss Prot:TCPB_HUMAN]	57/57	6.0/6.5	8-12/14-14	503-519	121-121	39-40
852	No identified protein		/51	/5.2				
877	No identified protein		/49	/7.0				
918	ANKRD26-like family C member 1A	[Swiss Prot:A26CA_HUMAN]	121/47	5.8/5.7	3-4/6-6	242-251	97-107	9-7
	Actin cytoplasmic 1	[Swiss Prot:ACTB_HUMAN]	42/47	5.3/5.7	5/10	390	131	53
	Actin cytoplasmic 2	[Swiss Prot:ACTG_HUMAN]	42/47	5.3/5.7	7/9	418	107	38
953	26S protease regulatory subunit 8	[Swiss Prot:PRS8_HUMAN]	46/46	7.1/7.6	9-2/15-7	180-53	41-31	45-21
	Mitochondrial import receptor subunit TOMM40 homolog	[Swiss Prot:TOM40_HUMAN]	38/46	6.8/7.6	1-1/2-4	48-66	48-38	7-15
	Fumarate hydratase mitochondrial precursor^b^	[Swiss Prot:FUMH_HUMAN]	55/46	8.9/7.6	1/3	61	61	7
1108	Nucleophosmin	[Swiss Prot:NPM_HUMAN]	33/39	4.6/5.1	3-4/5-5	83-203	39-65	24-25
1216	Annexin A2	[Swiss Prot:ANXA2_HUMAN]	39/35	7.6/8.0	11-13/8-10	650-265	99-51	45-38

^a^MW: molecular weight, pI: isoelectric point, ANKRD26: ankyrin repeat domain-containing protein 26, TOMM40: translocase of outer mitochondrial membrane 40 homolog (yeast). ^b^Only one peptide of the protein was recognised by matrix-assisted laser desorption ionization time-of-flight/time-of-flight mass spectrometry; identification spectrum for each protein spot is given in Additional file 5;^c^indicate number of unique identified peptides in MSMS and in MS+MSMS searches; ^d^identification was performed twice. When available, both values are given.

### IgG reactivity against HUVEC protein extracts

**Figure 4:**
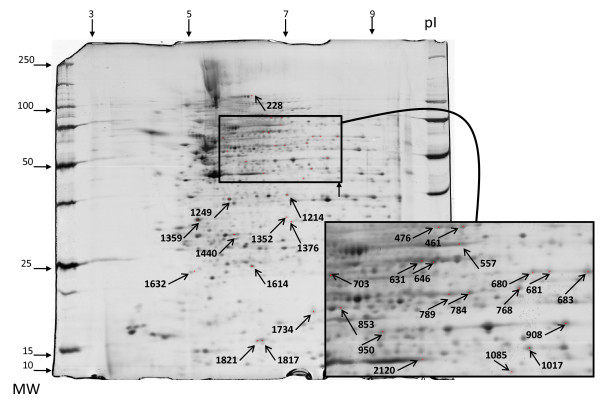
**Two-dimensional silver-stained protein pattern of total protein extracted from human umbilical vein endothelial cells**. Localisation of the 30 reactive spots recognised by two-thirds of the pools of sera from giant cell arteritis patients. Numbers were arbitrarily assigned by a computer program. Inset: Enlarged area ranging from 5.5 to 8.2 isoelectric points and 45 to 90 kDa. MW: molecular weight, pI: isoelectric points.

**Figure 5:**
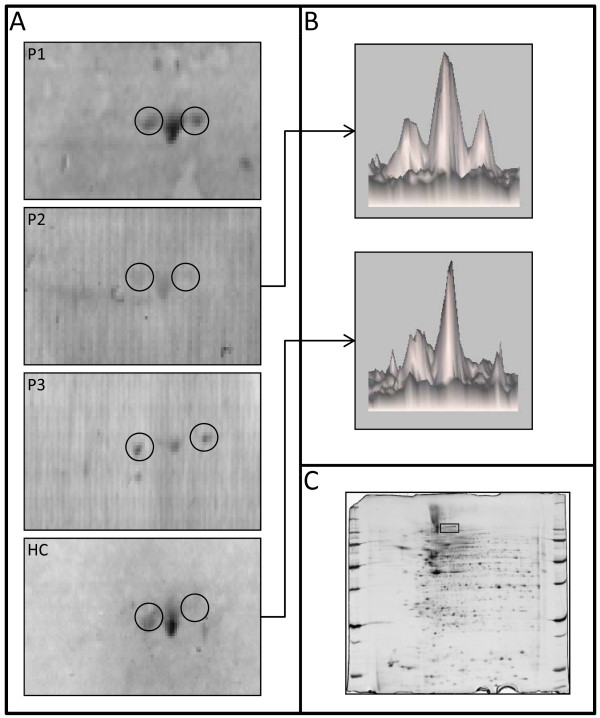
**Serum IgG reactivity to vinculin in serum of patients with giant cell arteritis**. **(A) **IgG reactivities to vinculin in three different pools of sera from three GCA patients each (P1 to P3) and one pool from twelve healthy controls (HC). **(B) **Vinculin spots are expressed in 3-D views for one representative sera pool of patients (top) and the pool of HCs (bottom). **(C) **Proteome of human umbilical vein endothelial cells showing the localisation of vinculin spots displayed in **(A)**.

The proteome of HUVECs contains 820 different proteins ranging from 3 to 10 IP and from 10 to 250 kDa. Among these, a mean (± SD) of 515 ± 73 protein spots were successfully detected after transfer onto PVDF membranes. Serum IgG from the HC pool recognised 79 protein spots, whereas IgG from the 3 pools of GCA patients recognised a mean (± SD) of 162 ± 3 protein spots corresponding to 191 different protein spots. Most of these 191 protein spots were recognised in only 1 pool of IgG from GCA patients and/or were also recognised in the HC pool. Among these protein spots, 45 were recognised in at least two-thirds of pools from GCA patients, including 30 that were not recognised in the HC pool (Additional file 7, Supplemental Table S3). Of these 30 proteins, 22 were identified by matrix-assisted laser desorption ionization time-of-flight/time-of-flight MS. Complete MS data are shown in Table 2. Localisations of identified protein spots in the analytical gel are depicted in Figure 4. Overall, three proteins were recognised by IgG in sera from GCA patients in HUVEC and VSMC protein extracts: mitochondrial fumarate hydratase, lamin A/C and vinculin. IgG reactivity against vinculin and lamin A/C in sera from GCA patients and the HC pool are depicted in Figure 5 and Additional file 8 respectively.

**Table 2:** Mass spectrometry data of the endothelial cell protein spots identified as specific target antigens^a^

Spot ID	Protein	SwissProt accession number	Theoretical/estimated MW, kDa	Theoretical/estimated pI	Number of unique identified peptides^c^	Total ion score	Best ion score	Sequence coverage, %
228	Vinculin	[Swiss Prot:VINC_HUMAN]	124/116	5.5/6.6	3/13	53	34	15
461	Lamin A/C	[Swiss Prot:LMNA_HUMAN]	74/80	6.6/7.3	11/23	573	82	39
	Semaphorin-4D precursor	[Swiss Prot:SEM4D_HUMAN]	96/80	8.3/7.3	2/2	44	29	2
476	Ezrin	[Swiss Prot:EZRI_HUMAN]	69/79	5.9/7.0	2/8	92	58	13
	Moesin	[Swiss Prot:MOES_HUMAN]	67/79	6.1/7.0	3/12	186	96	19
	Lamin A/C	[Swiss Prot:LMNA_HUMAN]	74/79	6.6/7.0	8/21	314	70	37
	Radixin	[Swiss Prot: RADI_HUMAN]	68/79	6.0/7.0	2/6	92	58	10
	Semaphorin-4D precursor	[Swiss Prot:SEM4D_HUMAN]	96/79	8.3/7.0	2/2	35	20	2
557	Far upstream element-binding protein 1	[Swiss Prot:FUBP1_HUMAN]	67/75	7.2/7.2	3/7	114	47	13
631	Lamin A/C	[Swiss Prot:LMNA_HUMAN]	74/71	6.6/6.9	6/10	184	48	15
646	Lamin A/C	[Swiss Prot:LMNA_HUMAN]	74/70	6.6/7.0	12/28	482	71	46
680	No protein identified		/66	/8.0				
681	No protein identified		/66	/8.2				
683	No protein identified		/66	/8.6				
703	No protein identified		/65	/5.9				
768	No protein identified		/60	/7.9				
784	Dihydrolipoyl dehydrogenase, mitochondrial precursor	[Swiss Prot:DLDH_HUMAN]	54/59	7.6/7.3	2/2	42	22	5
789	Inosine 5'-monophosphate dehydrogenase 2	[Swiss Prot:IMDH2_HUMAN]	56/58	6.4/7.1	4/7	169	94	17
853	No protein identified		/54	/6				
908	α-enolase	[Swiss Prot:ENOA_HUMAN]	47/50	7.0/8.3	7/12	450	143	47
950	Tripeptidyl peptidase 1 precursor	[Swiss Prot:TPP1_HUMAN]	61/50	6.0/6.4	3/5	89	34	15
1017	Fumarate hydratase, mitochondrial precursor	[Swiss Prot:FUMH_HUMAN]	55/48	8.9/8.0	6/7	243	71	24
1085	Heterogeneous nuclear ribonucleoprotein D0	[Swiss Prot:HNRPD_HUMAN]	38/43	7.6/7.8	3/3	122	69	11
1214	PDZ and LIM domain protein 1	[Swiss Prot:PDLI1_HUMAN]	36/37	6.6/7.4	5/10	269	62	44
1249	60S acidic ribosomal protein P0	[Swiss Prot:RLA0_HUMAN]	34/37	5.7/6.0	2/5	56	35	21
1352	Voltage-dependent anion-selective channel protein 2	[Swiss Prot:VDAC2_HUMAN]	32/33	7.5/7.4	4/4	155	75	18
1359	Annexin A5	[Swiss Prot:ANXA5_HUMAN]	36/33	4.9/5.3	10/12	538	86	52
1376	No protein identified		/32	/7.5				
1440	Heat shock protein β1	[Swiss Prot:HSPB1_HUMAN]	23/29	6.0/6.2	5/8	382	138	47
	NADH dehydrogenase [ubiquinone] iron-sulphur protein 3, mitochondrial precursor	[Swiss Prot:NDUS3_HUMAN]	30/29	7.0/6.2	2/6	70	38	26
1614	Protein DJ-1	[Swiss Prot:PARK7_HUMAN]	20/25	6.3/6.6	5/5	202	75	51
1632	No protein identified		/23	/5.3				
1734	Peptidyl-prolyl *cis-trans *isomerase A	[Swiss Prot:PPIA_HUMAN]	18/18	7.7/8	3/5	78	48	36
1817	Thioredoxin-dependent peroxide reductase, mitochondrial precursor	[Swiss Prot:PRDX3_HUMAN]	28/15	7.7/6.8	5/6	211	61	44
1821	Fatty acid-binding protein, epidermal^b^	[Swiss Prot:FABP5_HUMAN]	15/15	6.6/6.7	1/5	26	26	47
2120	Elongation factor Tu, mitochondrial precursor	[Swiss Prot:EFTU_HUMAN]	50/45	7.3/6.9	5/6	120	58	15
	Poly(rC)-binding protein 1^b^	[Swiss Prot:PCBP1_HUMAN]	37/46	6.7/6.9	1/2	36	36	6
	Heterogeneous nuclear ribonucleoprotein D0^b^	[Swiss Prot:HNRPD_HUMAN]	38/45	7.6/6.9	1/4	39	36	10

^a^MW: molecular weight, PDZ and LIM domain protein 1: postsynaptic density 95 (PSD95), pI: isoelectric point. ^b^Only one peptide of the protein was recognized by matrix-assisted laser desorption ionization time-of-flight/time-of-flight mass spectrometry; identification spectrum for each protein spot is given in Additional file 5. ^c^indicate number of unique identified peptides in MSMS and in MS+MSMS searches.

### Biological network analysis of identified autoantibody specificities

**Figure 6:**
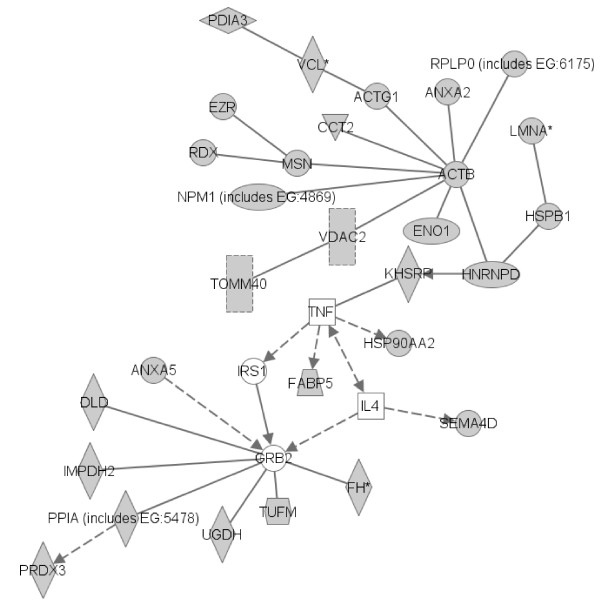
**Pathway generated by ingenuity pathway analysis with proteins identified as specific target antigens**. Solid lines indicate direct interactions. Dashed lines indicate indirect interactions. Arrows indicate stimulation. Mass spectrometry-identified proteins are depicted in grey. ACTB: actin, β; ACTG1: actin, γ1; ANXA2: annexin A2; ANXA5: annexin A5; CCT2: chaperonin containing TCP1, subunit 2 (β); DLD: dihydrolipoamide dehydrogenase; ENO1: enolase 1, α; EZR: ezrin; FABP5: fatty acid-binding protein 5; FH: fumarate hydratase; GRB2: growth factor receptor-bound protein 2; HNRNPD: heterogeneous nuclear ribonucleoprotein D; HSP90AA2: heat shock protein 90 α (cytosolic), class A member 2; HSPB1: HSPB1: heat shock 27-kDa protein 1; IL4: interleukin 4; IMPDH2: IMP (inosine 5'-monophosphate) dehydrogenase; IRS1: insulin receptor substrate 1; KHSRP: KH-type splicing regulatory protein = FUBP2; LMNA: lamin A/C; MSN: moesin; NPM1: nucleophosmin (nuclear phosphoprotein B23, numatrin); PDIA3: protein disulphide isomerase, family A, member 3; PPIA: peptidylpropyl isomerase A; PRDX3: peroxiredoxin 3; RDX: radixin; RPLPO: ribosomal protein, large, P0; SEMA4D: Sema domain, immunoglobulin domain (Ig), transmembrane domain (TM) and short cytosolic domain (semaphorin) 4D; TNF: tumour necrosis factor; TOMM40: translocase of outer mitochondrial membrane 40 homolog (yeast); TUFM: Tu translation elongation factor, mitochondrial; UGDH: UDP-glucose 6-dehydrogenase; VCL: vinculin; VDAC2: voltage-dependent anion channel 2.

Lists of VSMC and HUVEC proteins specifically recognised and/or recognised with high intensity by IgG in sera from GCA patients were analysed with IPA. Interestingly, most of the VSMC and HUVEC proteins specifically recognised and/or recognised with high intensity interacted with growth factor receptor-bound protein 2 (Grb2), a protein involved in VSMC proliferation. Therefore, we could depict the signalling network between HUVEC and VSMC proteins identified as major targets of autoantibodies in patients with GCA (Figure 6). Interestingly, TNF-α, IL-4 (Figure 6) and other molecules such as platelet-derived growth factor and IFN-γ (Additional file 9) were also involved in this signalling network.

## Discussion

In the present work, we detected IgG antibodies directed against the proteome of VSMCs and HUVECs in the sera of patients with GCA and identified their target antigens by using a 2-D immunoblotting technique and MS.

Few studies have focused on perturbations of the humoral immune system in patients with GCA. Few B lymphocytes are detected in temporal artery biopsies from patients with GCA [30]. When present, they are mainly found in the adventitial layer [31]. Moreover, plasma cells can be found in the adventitia in 7% to 24% of temporal artery biopsies from patients with GCA [32]. Plasma cells might localise in adventitia because of an infectious agent initiating vascular inflammation. However, a number of studies failed to identify an infectious agent, either a virus or bacteria, in the arterial wall by immunohistochemistry or PCR [33]. Alternatively, an autoantigen present in the arterial wall might trigger a specific immune response in GCA.

AECAs have been detected in healthy individuals [34] and in a number of systemic autoimmune diseases [10,35]. AECAs have been associated with disease activity in patients with vasculitis, particularly in those with anti-ANCA-associated vasculitis, Takayasu's arteritis or GCA [15], although these data remain controversial [36]. In addition, AECA could induce EC apoptosis in patients with systemic sclerosis [37]. However, the pathogenic role of AECAs has not yet been documented in GCA, and further investigations are necessary in this clinical setting.

Although to our knowledge anti-VSMC antibodies have not yet been reported in a human disease, such antibodies have been identified in a mouse model of vasculitis. Baiu *et al*. [17] showed that splenic mouse lymphocytes cultured with syngenic VSMCs induced vasculitic lesions after adoptive transfer into these mice. Serum collected from mice with vasculitis contained antibodies directed against VSMCs. Both wild-type and B-cell-deficient mice showed vascular inflammation after serum transfer, but mice deficient in both B and T cells (Rag2^-/-^) Yes it should did not, which suggests that immunoglobulin and cell-mediated pathways, particularly T cells, work in concert to contribute to the vasculitis lesions in this model. Thus, autoantibodies targeting proteins in the proteome of VSMCs might play a role in the pathogenesis of GCA, and their function needs to be further explored.

Few studies have been conducted to identify the potential targets of autoantibodies in GCA. Screening antigens in a cDNA library derived from normal human testis revealed high-intensity serum IgG reactivity directed against a number of ubiquitous autoantigens, including human lamin C, cytokeratin and mitochondrial cytochrome oxidase subunit II in the sera of patients with GCA [38]. Interestingly, we identified vinculin, lamin A/C and mitochondrial fumarate hydratase as target antigens of antibodies to proteins in the proteome of VSMCs and HUVECs. Vinculin is a cytoskeleton protein involved in extracellular matrix adhesion and intercellular junctions by binding to actin filaments. This protein has several interaction sites with numerous binding partners, including α-actin [39]. Changes in the relative content of vinculin and α-actin have been reported in the human aortic intima of patients with atherosclerosis [40]. Because vascular remodelling may occur in atherosclerosis, this type of change could be associated with vascular remodelling in GCA. Lamin A and C are both encoded by the *LMNA *gene and represent major constituents of the inner nuclear membrane. Mutations in the *LMNA *gene have been identified in a number of conditions, including Hutchinson-Gilford progeria syndrome [41]. The most frequent mutation responsible for progeria creates a truncated progeria mutant lamin A (progerin), which accumulates within the nuclei of human vascular cells and may be directly responsible for vascular involvement in progeria [42]. Other *LMNA *gene mutations, such as Dunnigan-type familial partial lipodystrophy (FPLD2), can lead to proatherogenic metabolic disturbances such as dyslipidemia, hyperinsulinemia, hypertension and diabetes. Premature atherosclerosis-induced FPLD2 seems to be associated with monogenic insulin resistance syndrome [43]. Identification of lamin A/C as target antigens in sera from patients with GCA seems interesting. Further investigations are necessary to characterise the implications of lamin A/C in vascular remodelling in this condition.

We found another antigen, far upstream element-binding protein 2 (FUBP = KHSRP), to be recognised by all five pools of sera from GCA patients in VSMC protein extracts. This protein, which binds to a DNA region called far-upstream element (FUSE), is a transcriptional activator of c-*myc*, a proto-oncogene that plays a key role in the regulation of cell growth, proliferation and differentiation. The FUSE-binding protein (FUBP) regulates FUSE activity [44]. Anti-FUBP antibodies were identified in synoviocytes in patients with rheumatoid arthritis, a condition marked by a proliferation of synovial tissue [45]. However, the potential pathogenic role of these antibodies has not yet been identified.

By using IPA, we found that most of the VSMC and HUVEC proteins specifically recognised and/or recognised with high intensity by IgG in sera from GCA patients interacted with Grb2. Grb2 is an intracellular linker protein that facilitates the activation of the small GTPase Ras by receptor tyrosine kinases and is involved in VSMC proliferation. Zhang and colleagues [46] reported that Grb2 is required for the development of neointima in response to vascular injury. Thus, Grb2 might be overexpressed and/or activated in ECs and VSMCs of patients with GCA and might stimulate the remodelling process. Moreover, proteins overexpressed in the remodelling process in the presence of activated Grb2 might trigger a specific immune response, possibly through structural antigen modifications occurring in the presence of metalloproteases and/or reactive oxygen species. A number of the VSMC and HUVEC proteins specifically recognised and/or recognised with high intensity by IgG in sera from GCA patients interacted with TNF-α. This result is in agreement with the pathophysiology of GCA and the ongoing inflammatory process in the arterial wall.

The combined use of 2-DE and immunoblotting offers an interesting approach to the identification of target antigens of autoantibodies [25]. However, our work has several limitations. Fewer than 1,500 protein spots were stained in the reference gel of VSMC and HUVEC protein extracts, which is less than the total number of proteins contained in these cells. Therefore, a number of proteins were probably lost at each step of the technique, depending on their charge, molecular weight, subcellular localisation and/or abundance in the cell. In addition, as expected, none of the identified antigens represented cell surface proteins, because protein extraction for 2-DE does not permit identification of membrane proteins. Finally, our pools of sera were from three patients each because this number was sufficiently low to allow the detection of strong reactivity that would be present in the serum of a single individual [25]. However, we cannot rule out the possibility that a low-intensity reactivity specific to a given individual might not be detected by using this pooling approach.

## Conclusions

We provide evidence that IgG antibodies directed toward the proteome of VSMCs and HUVECs are present in the sera of patients with GCA. These antibodies recognise cellular targets that play key roles in cell biology and the maintenance of homeostasis. The potential pathogenic role of these antibodies should be further investigated.

## Abbreviations

ACR: American College of Rheumatology; AECA: anti-endothelial cell antibody; ANCA: antineutrophil cytoplasm antibody; bFGF: basic fibroblast growth factor; CSS: Churg-Strauss syndrome; EC: endothelial cell; EGF: epidermal growth factor; ELISA: enzyme-linked immunosorbent assay; FCS: foetal calf serum; FUBP2: far upstream element-binding protein 2; FUSE: far upstream element; GCA: giant cell arteritis; Grb2: growth factor receptor-bound protein 2; HC: healthy control; HUVEC: human umbilical vein endothelial cell; IFN-γ: interferon-γ; IL: interleukin; IP: isoelectrofocalisation point; IPA: ingenuity pathway analysis; IPKB: Ingenuity Pathway Knowledgebase; MPA: microscopic polyangiitis; MS: mass spectrometry; PMSF: phenylmethylsulphonyl fluoride; PVDF: polyvinylidene fluoride; RT: reverse transcriptase; TNF-α: tumour necrosis factor α; VSMC: vascular smooth muscle cell; WG: Wegener's granulomatosis.

## Competing interests

AR, HD and LM have applied for a patent related to the content of this article (Patent Procédé de diagnostic d'une vascularite FR0951205).

## Authors' contributions

AR carried out the immunoblotting and proteomic experiments, analysed the results and drafted the manuscript. HD carried out immunoblotting and proteomic experiments with AR, participated in the analysis of the results and edited the manuscript. KHL carried out 1-D immunoblotting experiments and participated in the drafting of the manuscript. CA conducted the inclusion of patients into the study, analysed the results and edited the manuscript. MCT participated in the study design and the analysis of the results and also edited the manuscript. NT participated in immunoblotting and proteomic experiments, participated in the analysis of the results and edited the manuscript. CF performed ingenuity pathway analysis, participated in the analysis of the results and edited the manuscript. CB performed proteomic analysis, participated in the analysis of the results and edited the manuscript. BW provided immortalised VSMCs, participated in the analysis of the results and edited the manuscript. LG provided sera from patients, participated in the study design and analysis of the results and also edited the manuscript. LM provided sera from patients, designed the experiments, analysed the results and drafted the manuscript. All authors read and approved the final manuscript.

## Supplementary Material

Additional file 1 **Supplemental file**. Detailed data concerning 2-D electrophoresis technique and mass spectrometry identification [47-49]. Click here for file

Additional file 2 **Supplemental Table S1**. Clinical and histological characteristics of 15 patients with giant cell arteritis. Click here for file

Additional file 3 **Supplemental Figure S1**. One-dimensional immunoblot IgG reactivity from giant cell arteritis patients with vascular smooth muscle cell proteins. Serum samples from three patients with GCA were tested, and sera from four patients with Wegener's granulomatosis (WG), two with Churg-Strauss syndrome (CSS) and two with microscopic polyangeitis (MPA) used as vasculitis controls, intravenous immunoglobulin (IVIg) as a positive control and PBS and sera from two healthy controls (HCs) as negative controls were immunoblotted at a dilution of 1:100 with a soluble extract of immortalised human mammary artery VSMCs. Click here for file

Additional file 4 **Supplemental Table S2**. Antigens specifically recognised by IgG of three-fifths of the pools of sera from giant cell arteritis patients. Click here for file

Additional file 5 **Mass spectrometry data of target antigens recognised by only one peptide**. Click here for file

Additional file 6 **Supplemental Figure S2. 2-D immunoblots of IgG reactivity to vinculin in sera from patients with giant cell arteritis**. Protein extract is from vascular smooth muscle cells (VSMCs). **(A) **IgG reactivities of five different pools of sera from three giant cell arteritis patients each (P1 to P5) and one pool from twelve healthy controls (HCs). **(B) **Vinculin spots are expressed in 3-D views for one representative sera pool of patients (top) and the HC pool (bottom). **(C) **Proteome of VSMCs showing the localisation of vinculin spots displayed in **(A)**. Click here for file

Additional file 7 **Supplemental Table S3**. Antigens specifically recognised by IgG of two-thirds of the pools of sera from giant cell arteritis patients. Click here for file

Additional file 8 **Supplemental Figure S3. Two-dimensional immunoblots of IgG reactivity to lamin in sera of patients with giant cell arteritis. Protein extract is from human umbilical vein endothelial cells (HUVECs)**. **(A) **IgG reactivities to lamin of three different pools of sera from three giant cell arteritis patients each (P1 to P3) and one pool from twelve healthy controls (HCs). **(B) **Lamin spots are expressed in 3-D views for one representative sera pool from patients (top) and the HC pool (bottom). **(C) **Proteome of HUVECs showing the localisation of lamin spots displayed in **(A)**. Click here for file

Additional file 9 **Supplement Figure S4**. Protein network generated by merging the two pathways involved in target antigens. Protein extracts are from human umbilical vein endothelial cells and vascular smooth muscle cells. Solid lines indicate direct interactions. Dashed lines indicate indirect interactions. Arrows indicate stimulation. ABCA2: ATP-binding cassette, subfamily A, (ABC1), member 2; ACTB: actin, β; ACTG1: actin, γ1; ANXA2: annexin A2; ANXA5: annexin A5; CCND2: cyclin D2; CCT2: chaperonin containing TCP1, subunit 2 (β); COPA: coatomer protein complex, subunit α; CPOX: coproporphyrinogen oxidase; DLD: dihydrolipoamide dehydrogenase; ENO1: enolase 1, α; EZR: ezrin; FABP5: fatty acid-binding protein 5; FH: fumarate hydratase; FUBP1: far upstream element (FUSE)-binding protein 1; GRB2: growth factor receptor-bound protein 2; HNRNPD: heterogeneous nuclear ribonucleoprotein D; HSP90AA2: heat shock protein 90 kDa α (cytosolic), class A, member 2; HSPB1: heat shock 27-kDa protein 1; IFNG: interferon γ; IKBKG: inhibitor of κ light polypeptide gene enhancer in B cells, kinase γ; IL4: interleukin 4; IMPDH2: IMP (inosine 5'-monophosphate) dehydrogenase; IRS1: insulin receptor substrate 1; KHSRP: KH-type splicing regulatory protein; LMNA: lamin A/C; MSN: moesin; NDUFS3: NADH dehydrogenase (ubiquinone) iron-sulphur protein 3, 30 kDa (NADH coenzyme Q reductase); NPM1: nucleophosmin (nuclear phosphoprotein B23, numatrin); PARK7: Parkinson's disease (autosomal recessive early onset) 7; PDGF BB: platelet-derived growth factor B dimer; PDIA3: protein disulphide isomerase, family A, member 3; PHB: prohibitin; PPIA: peptidylpropyl-isomerase A; PRDX3: peroxiredoxin 3; PSMC5: proteasome 26S subunit, ATPase, 5; RDX: radixin; RPLPO: ribosomal protein, large, P0; SEMA4D: Sema domain, immunoglobulin domain (Ig), transmembrane domain (TM) and short cytosolic domain (semaphorin) 4D; TNF: tumour necrosis factor; TOMM40: translocase of outer mitochondrial membrane 40 homolog (yeast); TP53: tumour protein P53; TPP1: tripeptidyl 1 peptidase; TUFM: Tu translation elongation factor, mitochondrial; UGDH: UDP-glucose 6-dehydrogenase; VCL: vinculin; VDAC2: voltage-dependent anion channel 2. Click here for file
