# Supplementary material for: Identification of target antigens of anti-endothelial cell and anti-vascular smooth muscle cell antibodies in patients with giant cell arteritis: a proteomic approach
Source: Arthritis Res Ther. 2011 Jun 28;13(3):R107. doi: 10.1186/ar3388 (PMC3218922; doi:10.1186/ar3388)
Supplement: Additional file 1 — Supplemental file. Detailed data concerning 2-D electrophoresis technique and mass spectrometry identification [47-49]. [file ar3388-S1.DOC]

**Identification of target antigens of anti-endothelial-cell and anti-vascular-smooth-muscle-cell antibodies in patients with giant cell arteritis: a proteomic approach**

Alexis Régent^1,2,3^, Hanadi Dib^1,2^, Kim H. Ly^1,2,4*^, Christian Agard^5*^, Mathieu C. Tamby^1,2^, Nicolas Tamas^1,2^, Babette Weksler^6^, Christian Federici^1,2^, Cédric Broussard^7^, Loïc Guillevin^2,3^, Luc Mouthon^1,2,3^.

*Both authors contributed equally to the work.

**Supplemental data**

**Material and Methods**

**1-D Immunoblotting**

Confluent cells were detached with use of 0.05% trypsin, 0.53 mM EDTA. Protein extract was obtained by use of a 125-mM Tris, pH 6.8, solution containing 4% sodium dodecyl sulfate (SDS), 1.45 M β-mercaptoethanol, 1 µg/ml aprotinin, 1 µg/ml leupeptin, 1 µg/ml pepstatin and 1 mM of PMSF. Protein extract was then sonicated 4 x 30 s and boiled. Aliquots of 120 µl solubilised proteins were separated by polyacrylamide gel electrophoresis on 10% gels (Bio-Rad, Hercules, CA), transferred onto nitrocellulose membranes by use of a semi-dry electroblotter (Model A, Ancos, Hojby, Denmark) and incubated with sera from patients (GCA, WG, MPA, CSS) or healthy donors at 1:100 dilution or with IVIg solution at 1:100 dilution overnight at 4°C by use of a Cassette Miniblot System (Immunetics Inc., Cambridge, MA, USA). Detection of IgG reactivity was as previously reported [47] with a γ-chain-specific secondary rabbit anti-human IgG antibody coupled to alkaline phosphatase. Immunoreactivities were revealed by use of nitroblue tetrazolium/5-bromo-4-chloro-3-indolyl-phosphate (NBT/BCIP) (Sigma-Aldrich, St. Louis, MO, USA). Quantification of immunoreactivities involved densitometry in reflective mode (Epson Perfection 1200S densitometer, Seiko Epson Corp., Nagano-ken, Japan). The membranes were then stained with colloidal gold (Protogold, Biocell, Cardiff, UK) and scanned again to quantify transferred proteins.

**2-D Electrophoresis**

Proteins were isoelectrofocused with pH range IPG strips on the Protean IEF Cell Isoelectric Focusing System (Bio-Rad). Frozen samples were thawed and diluted, to a final volume of 400 µl each, into IPG sample buffer containing 7 M ultrapure urea (VWR, Fontenay-Sous-Bois, France), 2 M thiourea (Sigma), 4% (v/v) Chaps (Sigma), 7.5% (v/v) Triton X100 (Sigma),0.04% (v/v) carrier ampholyte mixture matching the pH range 3–10 (Pharmalytes 3–10; Amersham Biosciences, Uppsala, Sweden) and Bromophenol Blue (Sigma). IPG strips were rehydrated and focused in an automated run in the ceramic strip holders with 9-h rehydration at 20◦C, the 12 h at 50 V, 1 h at 200V, 1 h at 1000 V, then 6 h at 10,000 V and 1 h at 10,000 V.

Before running the second dimension, the strips were equilibrated for 15 min in 10 mL of the first equilibration solution (51 mM Tris [Amersham Biosciences], 6 mM urea, 40% [v/v] glycerol, 52 mM SDS, 32.4 mM DTT), then for 20 min in the second equilibration solution (51 mM Tris, 6 mM urea, 40% [v/v] glycerol, 52 mM SDS, 86.5 mM iodoacetamide). The second dimension electrophoresis was carried out in a Laemmli system on 7–18% polycrylamide linear gradient gels (20 cm × 20 cm × 1.5 mm) with a 18.5% buffer solution containing 2.5 M acrylamide (Amersham Biosciences), 24.7 mM piperazine diacrylyl (PDA) (Bio-Rad), 0.375 M Tris-HCl (Amersham Biosciences), pH 8.8, 15% (v/v) glycerol (Sigma), 3.5 mM SDS (Amersham Biosciences), 0.05% (v/v) TEMED (BioRad), 1.6 mM APS (BioRad), and a 7.5% buffer solution containing 1.0 M acrylamide, 10 mM PDA, 0.375 M Tris-HCl pH 8.8, 3.5 mM SDS, bi-distilled water, 0.06% (v/v) TEMED, and 2.4 mM APS were mixed. The equilibrated IPG gels were sealed on top of the 2-D gel with 1% agarose containing 50 mM Tris-Cl (pH 6.8), 2% (w/v) SDS, 30% (v/v) glycerol, and bromophenol blue and cathode-running buffer (24.8 mM Tris, 192 mM glycine, and 0.1% (w/v) SDS) was added. Gels were run at 40 V constant for 1 h, then 80 V constant for 1 h and then at 120V for 21 h 15 min.

The gels were transferred onto a polyvinylidene fluoride (PVDF) (Millipore,Bedford, MA) membrane by semidry transfer (BioRad) at 320 mA for 1 h 30 min. After blocking the membranes with PBS-0.2% (v/v) Tween for 90 min, each membrane was incubated overnight at +4°C with one pool of sera (3 sera for patients and 12 sera for healthy controls) diluted 1:100. Membranes were then extensively washed before being incubated with a secondary rabbit anti-human Fc-γ specific antibody coupled to alkaline phosphatase (Dako) for 90 min at room temperature. Immunoreactivities were revealed by use of the NBT–BCIP substrate (Sigma). Specific reactivities were determined by densitometrically scanning the membranes (densitometer GS-800, Bio-Rad) with use of Quantity one software (Bio-Rad). The membranes were then stained with colloidal gold (Protogold; British Biocell International, Cardiff, UK) and subjected to a second densitometric analysis to record labelled protein spots for each gel.

**Gel Staining**

Analytical gels were stained with ammoniacal silver nitrate. Images of both the gels and the 2-D blots were acquired by use of a densitometer (GS-800; Bio-Rad) and the images were exported to the Image Master 2D Platinum 6 software (Amersham). The number of spots was determined. A stack of the 2-D blot images before and after colloidal gold staining was acquired by the matching algorithm according to the manufacturer’s recommendations. Specific labels were then manually linked to the protein spots probed by serum IgG on both images. The algorithm automatically propagated these labels from the image of the colloidal gold-stained 2-D blot to the images of both reference silver-stained and preparative gels.

**Protein Identification**

For preparative 2-D gels, 100 μg of proteins were loaded onto IPG strips. Gels were stained after electrophoresis with 0.2% (w/v) Coomassie Blue R-250 in 50% (v/v) methanol and 10% (v/v) acetic acid, and destained in 50% (v/v) methanol and 2% (v/v) acetic acid. The relevant spots were selected by comparing the 2-D blots with the silver-stained analytical gels and colloidal Coomassie blue-stained preparative gels. The spots were then excised, and in-gel trypsin digestion was performed as described [48] with slight modification.

**In-gel trypsin digestion**

In-gel digestion was carried out with trypsin as described by Shevchenko [48] with minor modifications and involved for all steps a Freedom EVO 100 digester/spotter robot (Tecan, Männedorf, CH). Spots were first de-stained two times with a mixture of 100 mM ammonium bicarbonate (ABC) and 50% acetonitrile (ACN) for 45 min at 22°C and then dried with use of 100% ACN for 15 min. Protein spots were then treated with 25 mM ABC containing 10 mM DTT for 1 h at 60°C and then alkylated by use of 55 mM iodoacetamide in 25 mM ABC for 30 min in the dark at 22°C. Gel pieces were washed twice with 25 mM ABC and finally shrunk two times with 100% ACN for 15 min and dried with 100% ACN for 10 min. Bands were completely dehydrated after 1 h at 60°C. Gel pieces were incubated with 13 µL Sequencing Grade Modified Trypsin (Promega, WI; 12.5 µg/mL in 40 mMABC-10% ACN pH 8.0) overnight at 40°C. After digestion, peptides were washed with 30 µL of 25 mM ABC, shrunk with 100% (v/v) ACN and extracted twice with a mixture of 50% (v/v) ACN-5% (v/v) formic acid (FA). Extracts were then dried by vacuum centrifuge (Eppendorf, Hamburg, Germany). Finally, peptides were desalted using C18-ZipTips (Millipore) and two elutions, first with 50% (v/v) ACN-5% (v/v) FA and then 80% (v/v) ACN-5% (v/v) FA. Pooled elutions were allowed to dry at room temperature.

**Protein identification by mass spectrometry**

For mass spectrometry (MS) and MS/MS analysis, peptides were redissolved in 4 µL CHCA (5 mg/mL in 50% (v/v) ACN-0.1% (v/v) TFA). One microliter and a half of each sample was spotted directly onto a MALDI plate (Applied Biosystems, Foster City, CA). Droplets were allowed to dry at room temperature. The sample analysis involved a MALDI-TOF-TOF 4800 mass spectrometer (Applied Biosystems). Spectra acquisition and processing involved use of the 4000 series explorer software (Applied Biosystems) version 3.5.28193 in positive reflectron mode at fixed laser fluency with low mass gate and delayed extraction. External plate calibration was by four calibration points spotted onto the four corners of the plate with a mixture of five external standards (PepMix 1, LaserBio Labs, Sophia Antipolis, France). Peptide masses were acquired by steps of 50 spectra for 900 to 4000 Da. MS spectra were summed from 1000 laser shots by an Nd-YAG laser operating at 355 nm and 200 Hz. After filtering tryptic-, keratin- and matrix-contaminant peaks, up to 15 parent ions were selected for subsequent MS/MS fragmentation according to mass range, signal intensity, S/N, and absence of neighboring masses in the MS spectrum. MS/MS spectra were acquired in 1-kV positive mode, and 1000 shots were summed in increments of 50. Database searching involved MASCOT 2.2 (MatrixScience, London, UK) [49] *via* GPS explorer software (Applied Biosystems) version 3.6 combining MS and MS/MS interrogations on human proteins from Swiss-Prot databank release 54.5, 17 253 entries (www.expasy.org). The search parameters were as follows: carbamidomethylation as a variable modification for cysteins and oxidation as a variable modification for methionines. Up to one missed tryptic cleavage was permitted and mass accuracy tolerance of 30 ppm for precursors and 0.3 Da for fragments were used for all tryptic mass searches. Positive identification was based on a MASCOT score above the significance level (*i.e*.< 5%). In the case of peptides match to multiple members of a protein family, the reported protein is the one with the highest number of peptide matches.

**Abbreviation :**

CSS : Churg-Strauss syndrome

EC : endothelial cells

GCA : giant cell arteritis

MPA : microscopic polyangiitis

MS : mass spectrometry

NBT/BCIP : nitroblue tetrazolium/5-bromo-4-chloro-3-indolyl-phosphate

PDA : piperazine diacrylyl

PMSF : phenylmethanesulfonylfluoride

PVDF : polyvinylidene fluoride

SDS : sodium dodecyl sulfate

WG : Wegener's granulomatosis
