# Supplementary material for: Identification of target antigens of anti-endothelial cell and anti-vascular smooth muscle cell antibodies in patients with giant cell arteritis: a proteomic approach
Source: Arthritis Res Ther. 2011 Jun 28;13(3):R107. doi: 10.1186/ar3388 (PMC3218922; doi:10.1186/ar3388)
Supplement: Additional file 2 — Supplemental Table S1. Clinical and histological characteristics of 15 patients with giant cell arteritis. [file ar3388-S2.DOC]

**Supplemental table S1:** Clinical and histological characteristics of 15 patients with giant cell arteritis.

|  | Pool 1 | | | Pool 2 | | | Pool 3 | | | Pool 4 | | | Pool 5 | | |
| --- | --- | --- | --- | --- | --- | --- | --- | --- | --- | --- | --- | --- | --- | --- | --- |
| Patient | 1 | 2 | 3 | 4 | 5 | 6 | 7 | 8 | 9 | 10 | 11 | 12 | 13 | 14 | 15 |
| Histology of TAB | + | + | + | + | + | + | + | + | + | - | + | - | + | + | + |
| Disease evolution* | D | D | D | D | D | D | D | D | D | D | Rel | Rel | D | Fl | D |
| steroid dosage (mg/d)* | 0 | 0 | 0 | 0 | 0 | 0 | 0 | 0 | 0 | 0 | 0 | 0 | 0 | 25 | 0 |

* at the time of blood sampling.

D: Diagnosis; F: Female; Fl: Flare; M: Male; Rel: Relapse; +: Evidence of giant cell arteritis; -: Normal or fibrous endarteritis; TAB: Temporal artery biopsy
