# Supplementary figures and images for: Identification of target antigens of anti-endothelial cell and anti-vascular smooth muscle cell antibodies in patients with giant cell arteritis: a proteomic approach
Source: Arthritis Res Ther. 2011 Jun 28;13(3):R107. doi: 10.1186/ar3388 (PMC3218922; doi:10.1186/ar3388)

## Slide 1
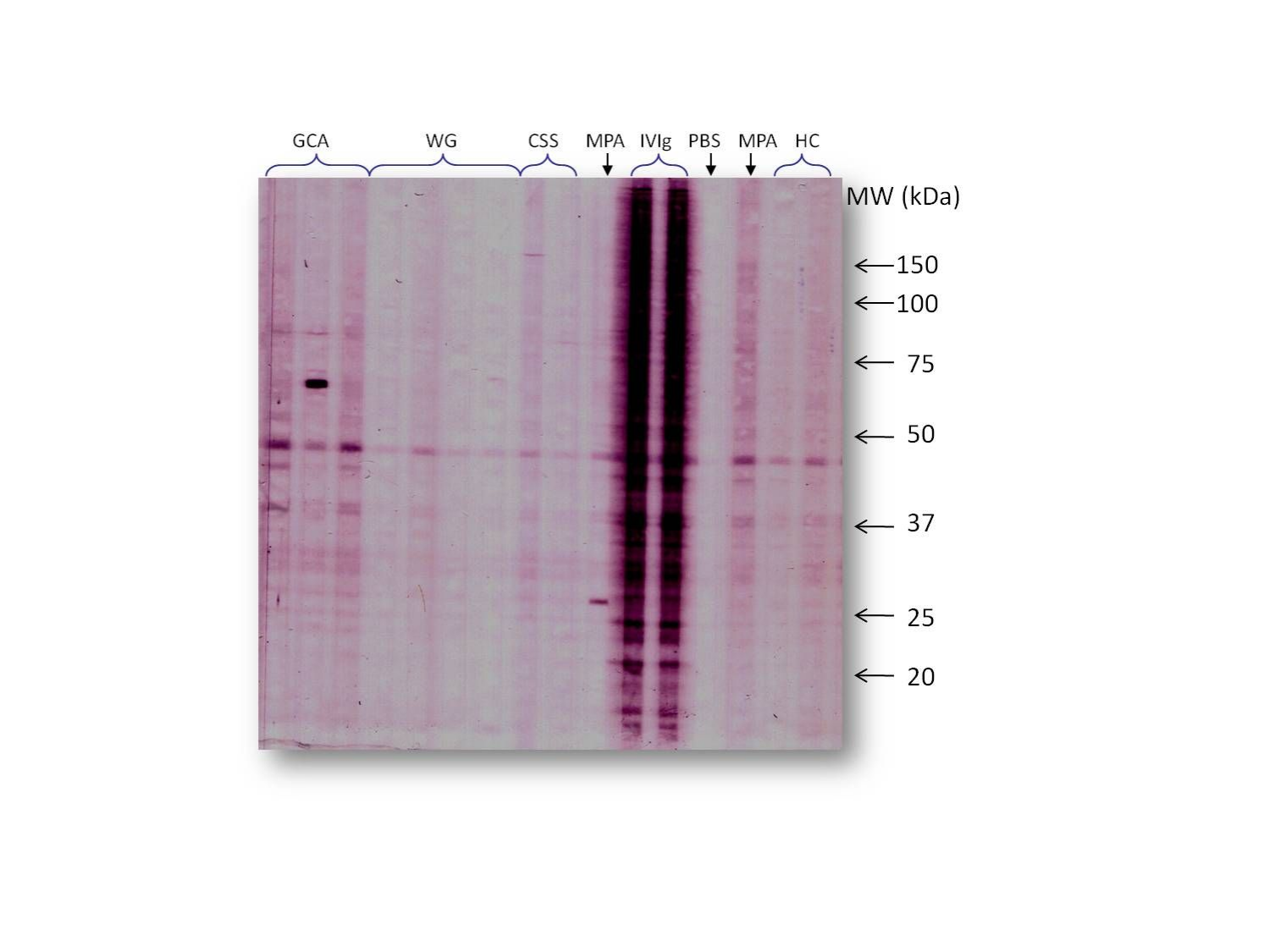

Supplement: Additional file 3 — Supplemental Figure S1. One-dimensional immunoblot IgG reactivity from giant cell arteritis patients with vascular smooth muscle cell proteins. Serum samples from three patients with GCA were tested, and sera from four patients with Wegener's granulomatosis (WG), two with Churg-Strauss syndrome (CSS) and two with microscopic polyangeitis (MPA) used as vasculitis controls, intravenous immunoglobulin (IVIg) as a positive control and PBS and sera from two healthy controls (HCs) as negative controls were immunoblotted at a dilution of 1:100 with a soluble extract of immortalised human mammary artery VSMCs. [file ar3388-S3.PPT]

## Slide 1
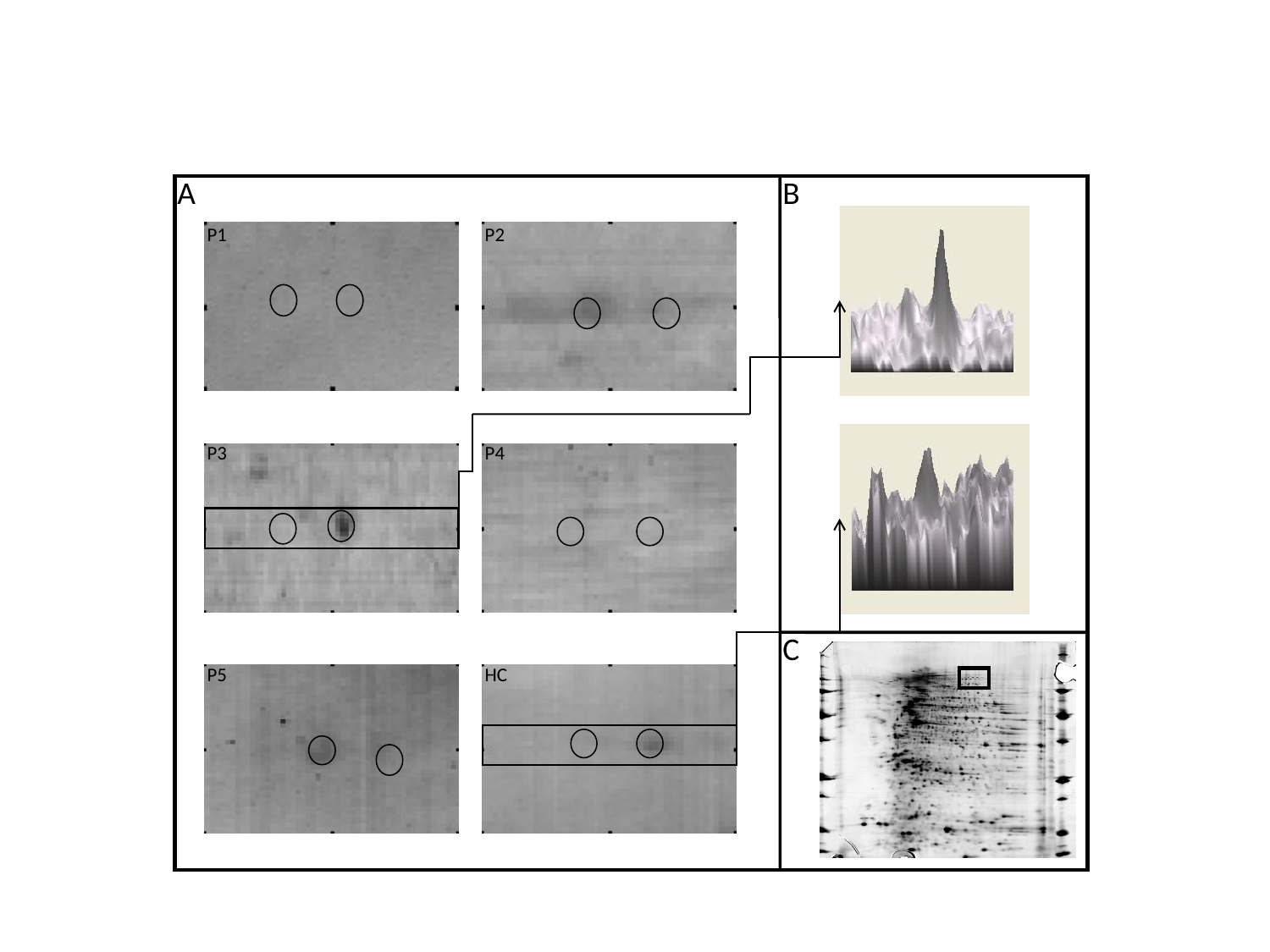

A
B
P1
P2
P3
P4
C
P5
HC

Supplement: Additional file 6 — Supplemental Figure S2. 2-D immunoblots of IgG reactivity to vinculin in sera from patients with giant cell arteritis. Protein extract is from vascular smooth muscle cells (VSMCs). (A) IgG reactivities of five different pools of sera from three giant cell arteritis patients each (P1 to P5) and one pool from twelve healthy controls (HCs). (B) Vinculin spots are expressed in 3-D views for one representative sera pool of patients (top) and the HC pool (bottom). (C) Proteome of VSMCs showing the localisation of vinculin spots displayed in (A). [file ar3388-S6.PPT]

## Slide 1
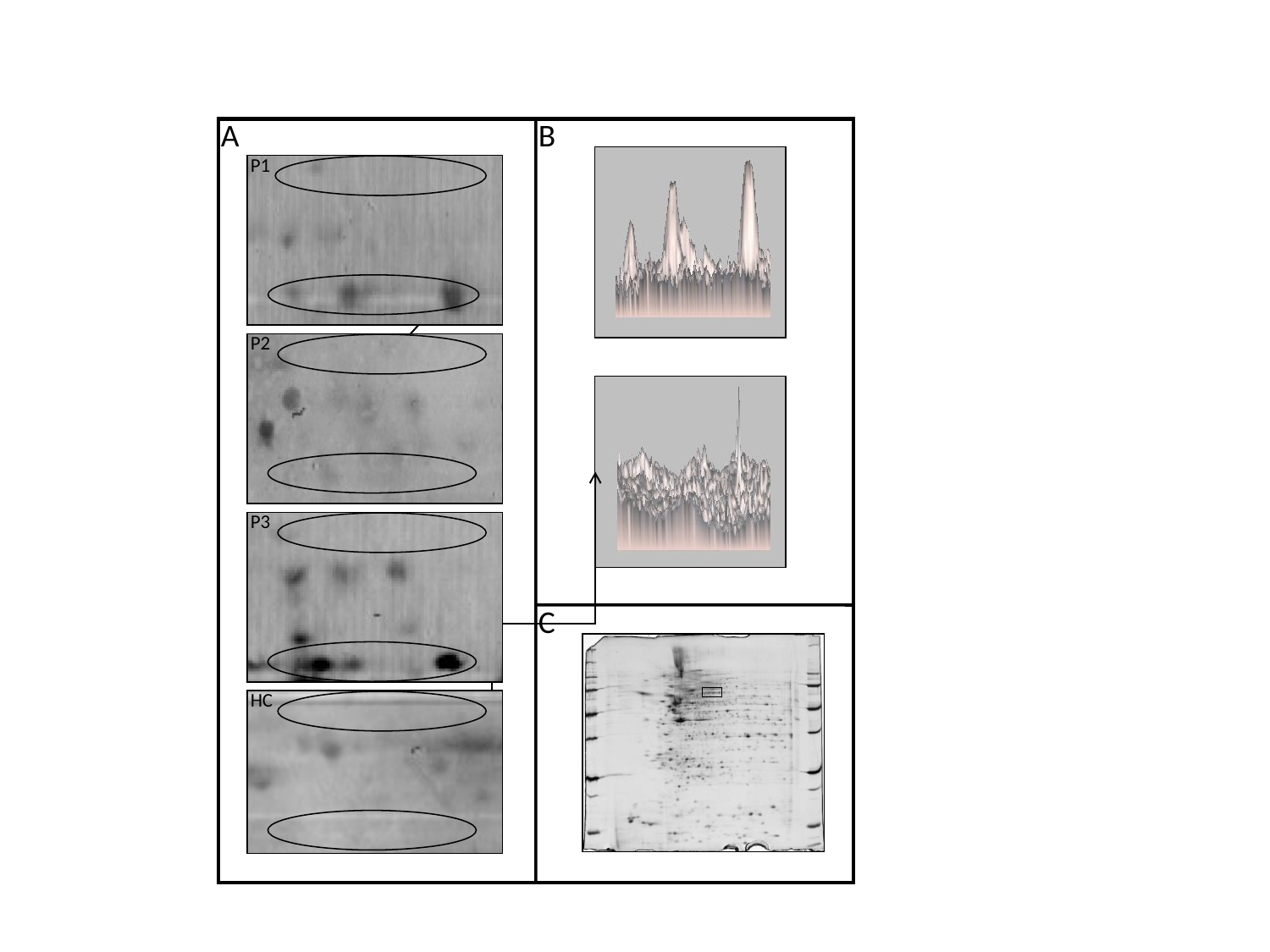

A
B
P1
P2
P3
C
HC

Supplement: Additional file 8 — Supplemental Figure S3. Two-dimensional immunoblots of IgG reactivity to lamin in sera of patients with giant cell arteritis. Protein extract is from human umbilical vein endothelial cells (HUVECs). (A) IgG reactivities to lamin of three different pools of sera from three giant cell arteritis patients each (P1 to P3) and one pool from twelve healthy controls (HCs). (B) Lamin spots are expressed in 3-D views for one representative sera pool from patients (top) and the HC pool (bottom). (C) Proteome of HUVECs showing the localisation of lamin spots displayed in (A). [file ar3388-S8.PPT]

## Slide 1
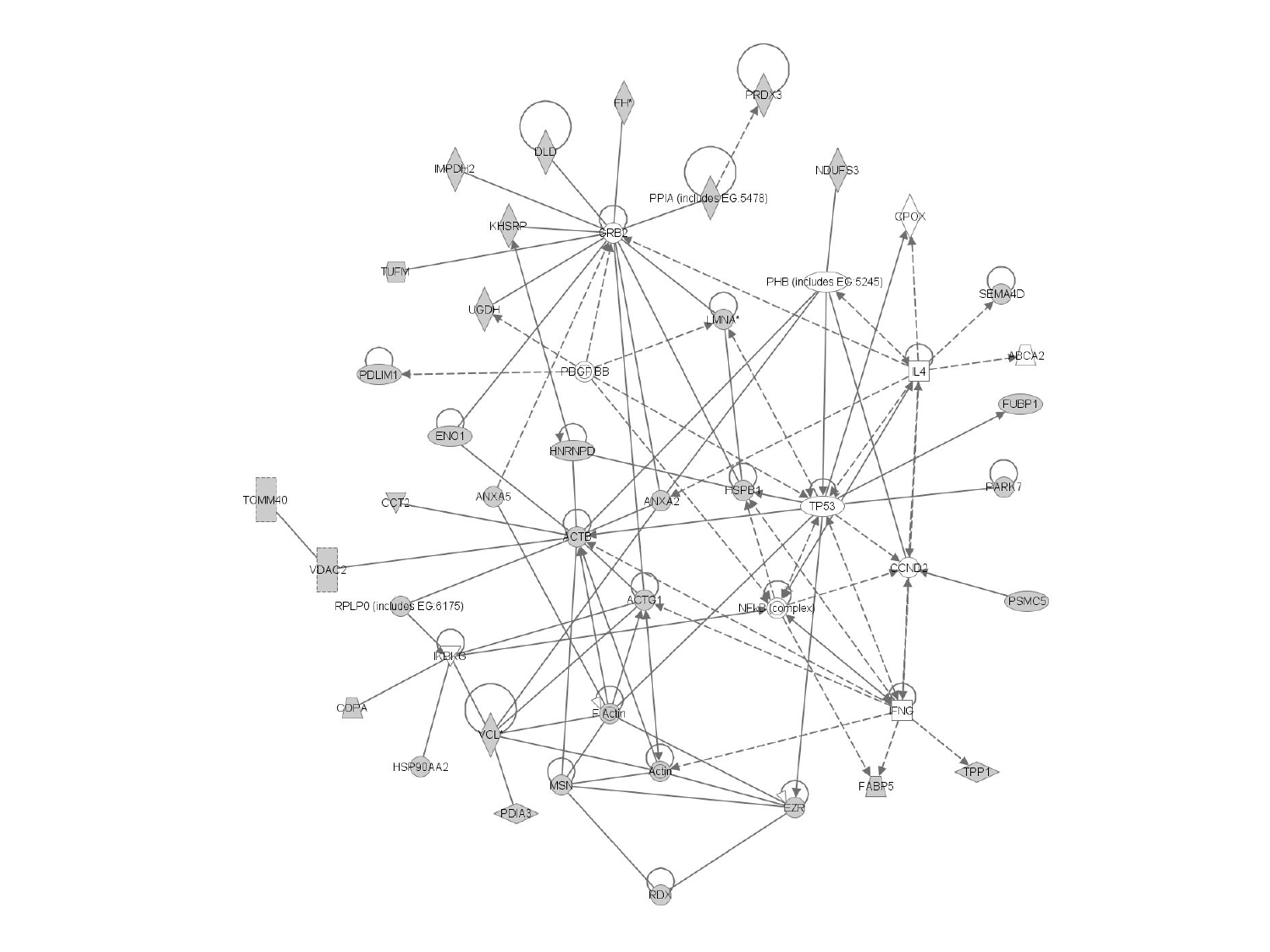

Supplement: Additional file 9 — Supplement Figure S4. Protein network generated by merging the two pathways involved in target antigens. Protein extracts are from human umbilical vein endothelial cells and vascular smooth muscle cells. Solid lines indicate direct interactions. Dashed lines indicate indirect interactions. Arrows indicate stimulation. ABCA2: ATP-binding cassette, subfamily A, (ABC1), member 2; ACTB: actin, β; ACTG1: actin, γ1; ANXA2: annexin A2; ANXA5: annexin A5; CCND2: cyclin D2; CCT2: chaperonin containing TCP1, subunit 2 (β); COPA: coatomer protein complex, subunit α; CPOX: coproporphyrinogen oxidase; DLD: dihydrolipoamide dehydrogenase; ENO1: enolase 1, α; EZR: ezrin; FABP5: fatty acid-binding protein 5; FH: fumarate hydratase; FUBP1: far upstream element (FUSE)-binding protein 1; GRB2: growth factor receptor-bound protein 2; HNRNPD: heterogeneous nuclear ribonucleoprotein D; HSP90AA2: heat shock protein 90 kDa α (cytosolic), class A, member 2; HSPB1: heat shock 27-kDa protein 1; IFNG: interferon γ; IKBKG: inhibitor of κ light polypeptide gene enhancer in B cells, kinase γ; IL4: interleukin 4; IMPDH2: IMP (inosine 5'-monophosphate) dehydrogenase; IRS1: insulin receptor substrate 1; KHSRP: KH-type splicing regulatory protein; LMNA: lamin A/C; MSN: moesin; NDUFS3: NADH dehydrogenase (ubiquinone) iron-sulphur protein 3, 30 kDa (NADH coenzyme Q reductase); NPM1: nucleophosmin (nuclear phosphoprotein B23, numatrin); PARK7: Parkinson's disease (autosomal recessive early onset) 7; PDGF BB: platelet-derived growth factor B dimer; PDIA3: protein disulphide isomerase, family A, member 3; PHB: prohibitin; PPIA: peptidylpropyl-isomerase A; PRDX3: peroxiredoxin 3; PSMC5: proteasome 26S subunit, ATPase, 5; RDX: radixin; RPLPO: ribosomal protein, large, P0; SEMA4D: Sema domain, immunoglobulin domain (Ig), transmembrane domain (TM) and short cytosolic domain (semaphorin) 4D; TNF: tumour necrosis factor; TOMM40: translocase of outer mitochondrial membrane 40 homolog (yeast); TP53: tumour protei [file ar3388-S9.PPT]
