# Supplementary material for: Identification of target antigens of anti-endothelial cell and anti-vascular smooth muscle cell antibodies in patients with giant cell arteritis: a proteomic approach
Source: Arthritis Res Ther. 2011 Jun 28;13(3):R107. doi: 10.1186/ar3388 (PMC3218922; doi:10.1186/ar3388)
Supplement: Additional file 4 — Supplemental Table S2. Antigens specifically recognised by IgG of three-fifths of the pools of sera from giant cell arteritis patients. [file ar3388-S4.DOC]

**Supplemental table S2. Antigens specifically recognized by IgG of three-fifths pools of sera from giant cell arteritis patients**. Protein extract is from vascular smooth muscle cell.

| Spot ID | Protein | GCA  pool 1 | GCA  pool 2 | | GCA  pool 3 | | GCA  pool 4 | | GCA  pool 5 | |
| --- | --- | --- | --- | --- | --- | --- | --- | --- | --- | --- |
| 173 | Vinculin |  | | + | | + | |  | | + |
| 294 | Putative HSP 90-alpha A2 | + | |  | |  | | + | | + |
| 340 | Far upstream element-binding protein 2 | + | | + | | + | | + | | + |
| 341 | Far upstream element-binding protein 2 | + | | + | | + | | + | |  |
| 344 | Far upstream element-binding protein 2 |  | | + | | + | | + | |  |
| 580 | Lamin A/C |  | | + | | + | |  | | + |
|  | Coatomer subunit alpha |  | | + | | + | |  | | + |
| 598 | UDP-glucose 6-dehydrogenase | + | | + | | + | |  | |  |
| 609 | No protein identified | + | | + | | + | |  | |  |
| 683 | No protein identified |  | | + | |  | | + | | + |
| 686 | Protein disulfide-isomerase A3 | + | | + | | + | |  | |  |
| 694 | Protein disulfide-isomerase A3 | + | | + | | + | |  | |  |
| 702 | No protein identified | + | | + | |  | | + | | + |
| 734 | T-complex protein 1 subunit beta | + | | + | |  | | + | |  |
| 852 | No protein identified | + | |  | |  | | + | | + |
| 877 | No protein identified | + | | + | | + | | + | |  |
| 918 | ANKRD26-like family C member 1A | + | | + | | + | |  | | + |
|  | Actin cytoplasmic 1 | + | | + | | + | |  | | + |
|  | Actin cytoplasmic 2 | + | | + | | + | |  | | + |
| 953 | 26S protease regulatory subunit 8 | + | |  | |  | | + | | + |
|  | Mitochondrial Import receptor subunit TOM40 homolog | + | |  | |  | | + | | + |
|  | Fumarate hydratase mitochondrial | + | |  | |  | | + | | + |
| 1108 | Nucleophosmin | + | | + | | + | |  | |  |
| 1216 | Annexin A2 | + | | + | | + | |  | |  |

ANKRD26: Ankyrin repeat domain-containing protein 26; GCA: giant cell arteritis; HSP: heat shock protein; ID: identity; UDP: Uridine diphosphate; TOM40 : Translocase of outer membrane 40kDa
