# Supplementary material for: Identification of target antigens of anti-endothelial cell and anti-vascular smooth muscle cell antibodies in patients with giant cell arteritis: a proteomic approach
Source: Arthritis Res Ther. 2011 Jun 28;13(3):R107. doi: 10.1186/ar3388 (PMC3218922; doi:10.1186/ar3388)
Supplement: Additional file 5 — Mass spectrometry data of target antigens recognised by only one peptide. [file ar3388-S5.PDF]

Supplemental File 2. Mass Spectrometry Data. The following information is given to highlight the MS/MS spectra for the 8 proteins identified with a single peptide showed in Tables 2 and 4.

Precursor 1348.667Da MS/MS results (protein ID 294 - putative HSP90lamine A/C)

Mascot Search Results

Match to: **H90B3_HUMAN** Score: **40**

Putative heat shock protein HSP 90-beta-3 OS=Homo sapiens GN=HSP90AB3P PE=5 SV=1

1 MPEEVHHGEE EVETFAFQAE IAQLISLIIN TFYSNEEIFL QELISNASDA
51 LDKIRYESLT DPSKLDSGKE LKIDIIPNPQ ERTLALVDTG IGMTKADLIN
101 NLRTIAKSGT KACMEALQAE KLVVITKHND DEQYAWESSA GGSFTVHADH
151 GEPIGRGTKV ILHLKEDQTE YLEERRVKEV VKKHSQFIGY PITLYLEKEQ
201 DKEISDDEAE EEKGEKEEED KDDEEKPKIK DVGSDEEDDS KEYGEFYKSL
251 TSDWEDHLAV KHFSVEGQLE FRALLFSPRR APFDLFENKK KKNNIKLYVR
301 RVFIMDSCDE LIPEYLNFIH GVVDSEDLPL NISREMLQQS KILKYVSHMK
351 ETQKSTYYIT GESKEQVANS AFVERVRKQG FEVVYMTEPI DEYCVQQLKE
401 FDGKSLVSVT KEGLELPEDE EEKKKMEESK EKFENLCKLM KEILDKKVEK
451 VTISNRLVSS PCCIVTSTYG WTANMEQIMK AQALRDNSTM GYMMAKKHLE
501 INPDHPIMET LRQKAEADKN DKAVKDLVVL LFETALLSSG FSLEDPQTHS
551 NHIYHMIKLG LGTDEDEVAA EEPSDAVPDE IPPLEGDEDA SRMEEVD

Start - End	Observed	Mr(expt)	Mr(calc)	ppm	Miss	Sequence
262 - 272	1348.667	1347.659	1347.657	2	0	K.HFSVEGQLEFR.A

(Ions score 40)

MS/MS Fragmentation of **HFSVEGQLEFR**
Found in **H90B3_HUMAN**, Putative heat shock protein HSP 90-beta-3 OS=Homo sapiens GN=HSP90AB3P PE=5 SV=1

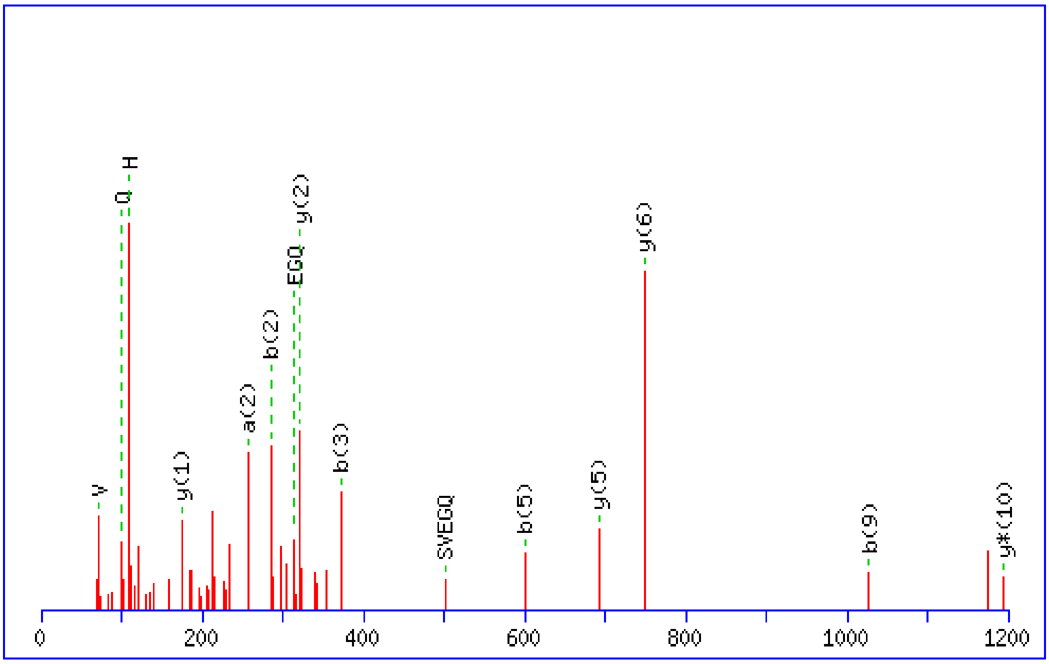

#	b	Seq.	y	#
1	138.066	H		11
2	285.135	F	1211.606	10
3	372.167	S	1064.537	9
4	471.235	V	977.505	8
5	600.278	E	878.437	7
6	657.299	G	749.394	6
7	785.358	Q	692.373	5
8	898.442	L	564.314	4
9	1027.484	E	451.23	3
10	1174.553	F	322.187	2
11		R	175.119	1

Precursor 1023.503Da MS/MS results (protein ID 580 - lamine A/C)

Mascot Search Results

Match to: **LMNA_HUMAN** Score: **38** Expect: **3.6**

Lamin-A/C OS=Homo sapiens GN=LMNA PE=1 SV=1

1 METPSQRRAT RSGAQASSTP LSPTRITRLQ EKEDLQELND

RLAVYIDRVR

51 SLETENAGLR LRITESEEVV SREVSGIKAA YEAELGDARK TLDSVAKERA

101 RLQLELSKVR EEFKELKARN TKKEGDLIAA QARLKDLEAL LNSKEAALST
151 ALSEKRTLEG ELHDLRGQVA KLEAALGEAK KQLQDEMLRR
VDAENRLQTM
201 KEELDFQKNI YSEELRETKR RHETRLVEID NGKQREFESR LADALQELRA
251 QHEDQVEQYK KELEKTYSAK LDNARQSAER NSNLVGAAHE
ELQQSRIRID
301 SLSAQLSQLQ KQLAAKEAKL RDLEDSLARE RDTSRRLLAE
KEREMAEMRA
351 RMQQQLDEYQ ELLDIKLALD MEIHAYRKLL EGEEERLRLS PSPTSQRSRG
401 RASSHSSQTQ GGGSVTKKRK LESTESRSSF SQHARTSGRV
AVEEVDEEGK
451 FVRLRNKSNE DQSMGNWQIK RQNGDDPLLT YRFPPKFTLK
AGQVVTIWAA
501 GAGATHSPPT DLVWKAQNTW GCGNSLRTAL INSTGEEVAM
RKLVRSVTVV
551 EDDEDEDGDD LLHHHHGSHC SSSGDPAEYN LRSRTVLCGT
CGQPADKASA
601 SGSGAQVGGP ISSGSSASSV TVTRSYRSVG GSGGGSFGDN
LVTRSYLLGN
651 SSPRTQSPQN CSIM

Start - End	Observed	Mr(expt)	Mr(calc)	ppm	Miss	Sequence
209 - 216	1023.503	1022.496	1022.503	-7	0	K.NIYSEELR.E **(Ions score 24)**

MS/MS Fragmentation of **NIYSEELR**
Found in **LMNA_HUMAN**, Lamin-A/C OS=Homo sapiens GN=LMNA PE=1
SV=1

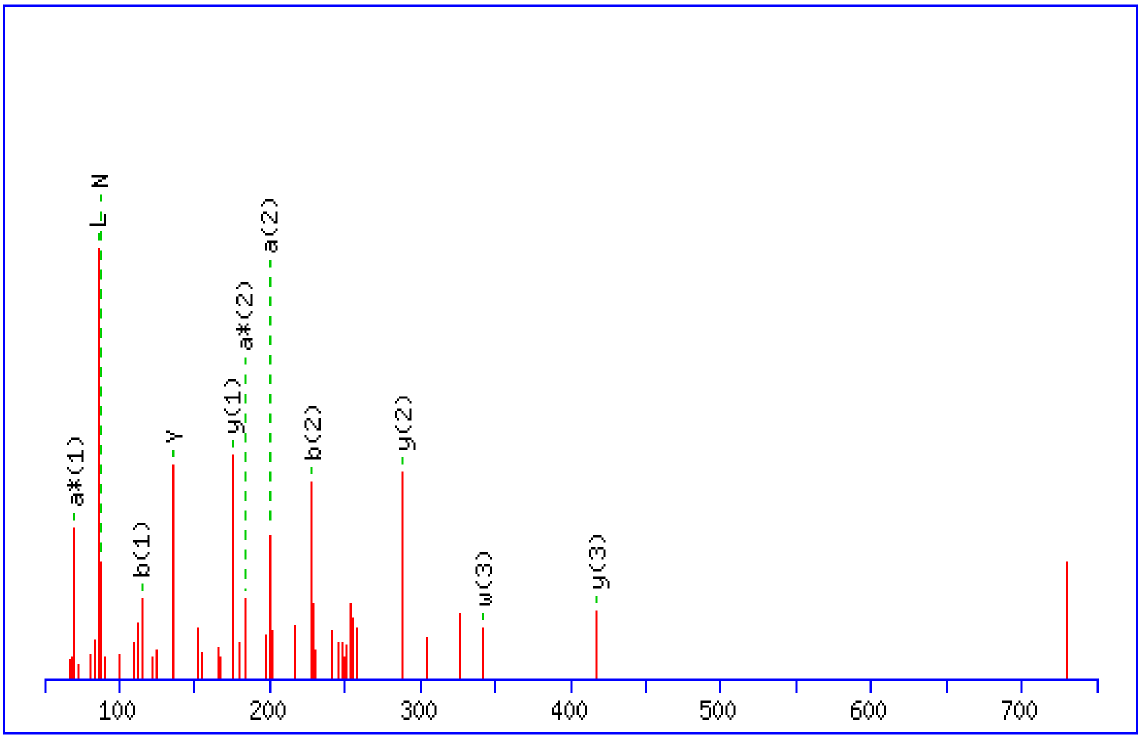

#	b	Seq.	y	#
1	115.05	N		8
2	228.134	I	909.468	7
3	391.198	Y	796.384	6
4	478.23	S	633.32	5
5	607.272	E	546.288	4
6	736.315	E	417.246	3
7	849.399	L	288.203	2
8		R	175.119	1

Precursor 1023.503Da MS/MS results (protein ID 580 - coatamer subunit-alapha)

Mascot Search Results

Match to: **COPA_HUMAN** Score: **37**

Coatomer subunit alpha OS=Homo sapiens GN=COPA PE=1 SV=2

1 MLTKFETKSA RVKGLSFHPK RPWILTSLHN GVIQLWDYRM
CTLIDKFDEH

51 DGPVRGIDFH KQQPLFVSGG DDYKIKVWNY KLRRCLFTLL GHLDYIRTTF

101 FHHEYPWILS ASDDQTIRVW NWQSRTCVCV LTGHNHYVMC
AQFHPTEDLV

151 VSASLDQTVR VWDISGLRKK NLSPGAVESD VRGITGVDLF GTTDAVVKHV

201 LEGHDRGVNW AAFHPTMPLI VSGADDRQVK IWRMNESKAW
EVDTCRGHYN

251 NVSCAVFHPR QELILSNSED KSIRVWDMSK RTGVQTFRRD
HDRFWVLAAH

301 PNLNLFAAGH DGGMIVFKLE RERPAYAVHG NMLHYVKDRF
LRQLDFNSSK

351 DVAVMQLRSG SKFPVFNMSY NPAENAVLLC TRASNLENST YDLYTIPKDA

401 DSQNPDAPEG KRSSGLTAVW VARNRFAVLD RMHSLLIKNL
KNEITKKVQV

451 PNCDEIFYAG TGNLLLRDAD SITLFDVQQK RTLASVKISK VKYVIWSADM

501 SHVALLAKHA IVICNRKLDA LCNIHENIRV KSGAWDESGV
FIYTTSNHIK

551 YAVTTGDHGI IRTLDLPIYV TRVKGNNVYC LDRECRPRVL TIDPTEFKFK

601 LALINRKYDE VLHMVRNAKL VGQSIIAYLQ KKGYPEVALH FVKDEKTRFS

651 LALECGNIEI ALEAAKALDD KNCWEKLGEV ALLQGNHQIV EMCYQRTKNF

701 DKLSFLYLIT GNLEKLRKMM KIAEIRKDMS GHYQNALYLG DVSERVRILK

751 NCGQKSLAYL TAATHGLDEE AESLKETFDP EKETIPDIDP NAKLLQPPAP

801 IMPLDTNWPL LTVSKGFFEG TIASKGKGGA LAADIDIDTV GTEGWGEDAE

851 LQLDEDGFVE ATEGLGDDAL GKGQEEGGGW DVEEDLELPP
ELDISPGAAG
901 GAEDGFFVPP TKGTSPTQIW CNNSQLPVDH ILAGSFETAM RLLHDQVGVI
951 QFGPYKQLFL QTYARGRTTY QALPCLPSMY GYPNRNWKDA
GLKNGVPAVG
1001 LKLNDLIQRL QLCYQLTTVG KFEEAVEKFR SILLSVPLLV VDNKQEIAEA
1051 QQLITICREY IVGLSVETER KKLPKETLEQ QKRICEMAAY FTHSNLQPVH
1101 MILVLRTALN LFFKLKNFKT AATFARRLLE LGPKPEVAQQ TRKILSACEK
1151 NPTDAYQLNY DMHNPFDICA ASYRPIYRGK PVEKCPLSGA
CYSPEFKGQI
1201 CRVTTVTEIG KDVIGLRISP LQFR

Start - End	Observed	Mr(expt)	Mr(calc)	ppm	Miss	Sequence
719 - 726	1023.503	1022.496	1022.525	-29	1	K.MMKIAEIR.K 2 __Oxidation (M) (Ions score 37)

MS/MS Fragmentation of **MMKIAEIR**
Found in **COPA_HUMAN**, Coatomer subunit alpha OS=Homo sapiens GN=COPA PE=1 SV=2

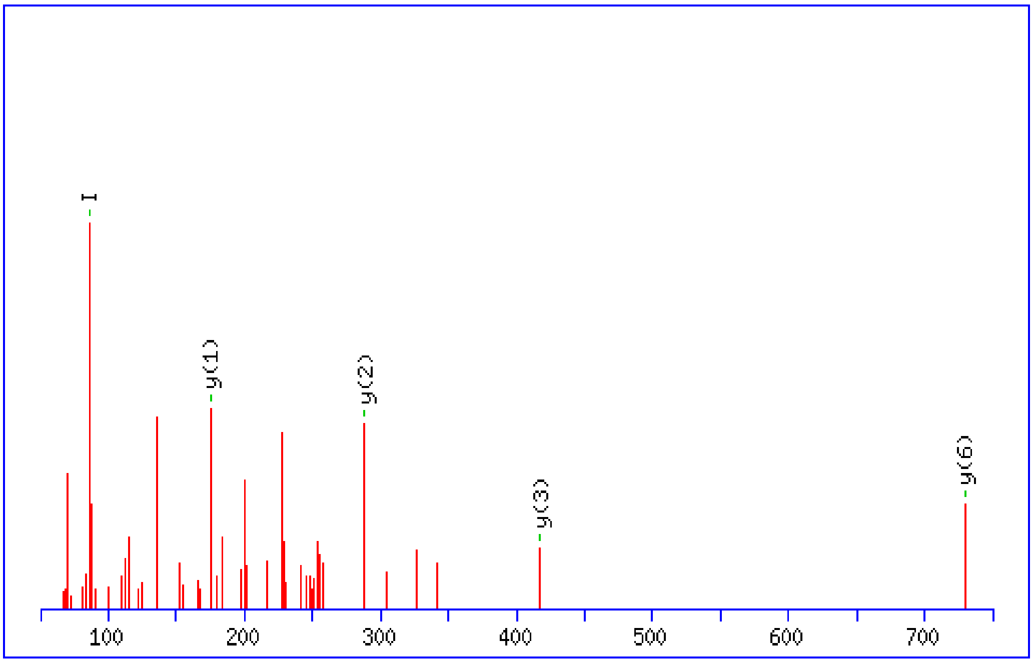

#	b	Seq.	y	#
1	84.044	M		8
2	167.082	M	812.499	7
3	295.176	K	**729.462**	6
4	408.261	I	601.367	5
5	479.298	A	488.283	4
6	608.34	E	**417.246**	3
7	721.424	I	**288.203**	2
8		R	**175.119**	1

Precursor 1370.688Da MS/MS results (protein ID 598 - UDP glucose 6 deshydrogenase)

Mascot Search Results

Match to: **UGDH_HUMAN** Score: **39**

UDP-glucose 6-dehydrogenase OS=Homo sapiens GN=UGDH PE=1 SV=1

Matched peptides shown in **Bold Red**

1 MFEIKKICCI GAGYVGGPTC SVIAHMCPEI RVTVVDVNES RINAWNSPTL
51 PIYEPGLKEV VESCRGKNLF FSTNIDDAIK EADLVFISVN TPTKTYGMGK
101 GRAADLKYIE ACARRIVQNS NGYKIVTEKS TVPVRAAESI RRIFDANTKP
151 NLNLQVLSNP EFLAEGTAIK DLKNPDR**VLI GGDETPEGQR** AVQALCAVYE
201 HWVPREKILT TNTWSSELSK LAANAFLAQR ISSINSISAL CEATGADVEE
251 VATAIGMDQR IGNKFLKASV GFGGSCFQKD VLNLVYLCEA LNLPEVARYW
301 QQVIDMNDYQ RRRFASRIID SLFNTVTDKK IAILGFAFKK DTGDTRESSS
351 IYISKYLMDE GAHLHIYDPK VPREQIVVDL SHPGVSEDDQ VSRLVTISKD
401 PYEACDGAHA VVICTEWDMF KELDYERIHK KMLKPAFIFD GRRVLDGLHN
451 ELQTIGFQIE TIGKKVSSKR IPYAPSGEIP KFSLQDPPNK KPKV

Start - End	Observed	Mr(expt)	Mr(calc)	ppm	Miss	Sequence
**178 - 190**	**1370.688**	**1369.681**	**1369.684**	**-2**	**0**	**R.VLIGGDETPEGQR.A (Ions score 39)**

MS/MS Fragmentation of **VLIGGDETPEGQR**
Found in **UGDH_HUMAN**, UDP-glucose 6-dehydrogenase OS=Homo sapiens GN=UGDH PE=1 SV=1

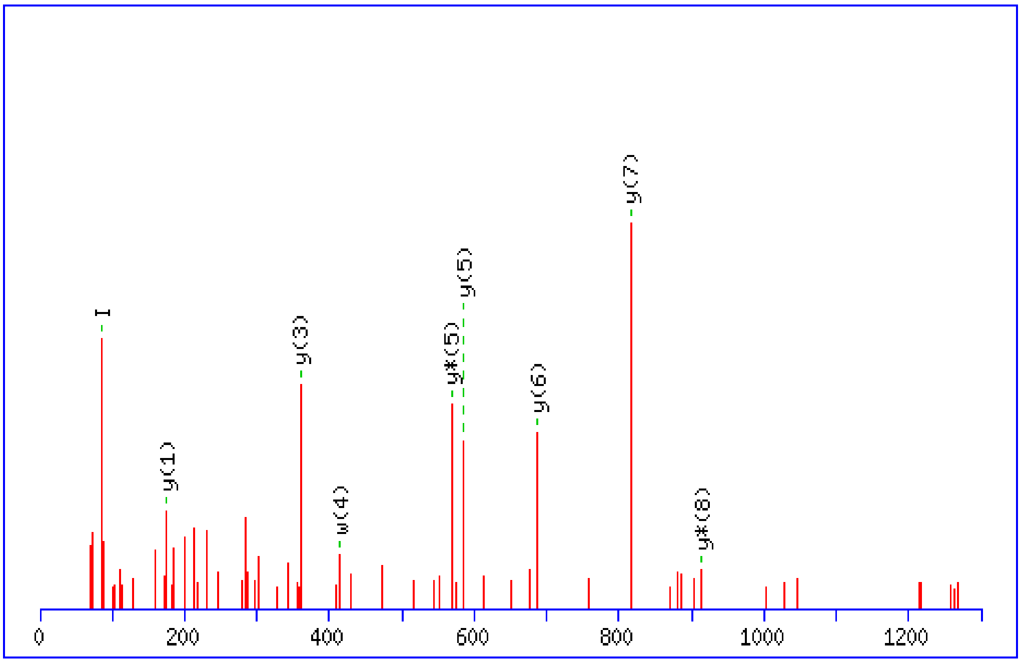

#	b	Seq.	y	#
1	100.076	V		13
2	213.16	L	1271.623	12
3	326.244	I	1158.539	11
4	383.265	G	1045.454	10
5	440.287	G	988.433	9
6	555.314	D	931.412	8
7	684.356	E	**816.385**	7
8	785.404	T	**687.342**	6
9	882.457	P	**586.294**	5
10	1011.499	E	489.242	4
11	1068.521	G	**360.199**	3
12	1196.579	Q	303.178	2
13		R	**175.119**	1

Precursor 1214.597Da MS/MS results (protein ID 953 - fumarate hydratase mitochondriale)

Mascot Search Results

Match to: **FUMH_HUMAN** Score: **61**

Fumarate hydratase, mitochondrial OS=Homo sapiens GN=FH PE=1 SV=3

Matched peptides shown in **Bold Red**

1 MYRALRLLAR SRPLVRAPAA ALASAPGLGG AAVPSFWPPN

AARMASQNSF

51 RIEYDTFGEL KVPNDKYYGA QTVRSTMNFK IGGVTERMPT PVIKAFGILK

101 RAAAEVNQDY GLDPKIANAI MKAADEVAEG KLNDHFPLVV

WQTGSGTQTN

151 MNVNEVISNR AIEMLGGELG SKIPVHPNDH VNKSQSSNDT FPTAMHIAAA

201 IEVHEVLLPG LQKLHDALDA KSKEFAQIIK IGRTHTQDAV PLTLGQEFSG

251 YVQQVKYAMT RIKAAMPRIY ELAAGGTAVG TGLNTRIGFA EKVAAKVAAL

301 TGLPFVTAPN KFEALAAHDA LVELSGAMNT TACSLMKIAN DIRFLGSGPR

351 SGLGELILPE NEPGSSIMPG KVNPTQCEAM TMVAAQVMGN

HVAVTVGGSN

401 GHFELNVFKP MMIKNVLHSA RLLGDASVSF TENCVVGIQA NTERINKLMN

451 ESLMLVTALN PHIGYDKAAK IAKTAHKNGS TLKETAIELG YLTAEQFDEW

501 VKPKDMLGPK

Start - End	Observed	Mr(expt)	Mr(calc)	ppm	Miss	Sequence
**52 - 61**	**1214.597**	**1213.590**	**1213.587**	**3**	**0**	**R.IEYDTFGELK.V**

(Ions score 61)

MS/MS Fragmentation of **IEYDTFGELK**

Found in **FUMH_HUMAN**, Fumarate hydratase, mitochondrial OS=Homo sapiens GN=FH PE=1 SV=3

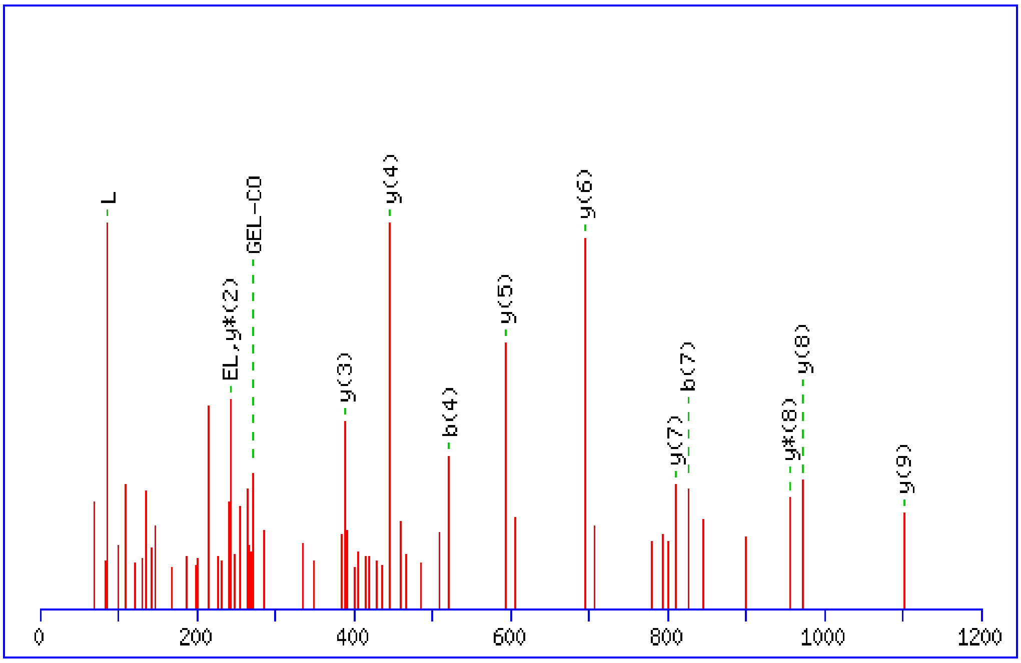

#	b	Seq.	y	#
1	114.091	I		10
2	**243.134**	E	**1101.51**	9
3	406.197	Y	**972.467**	8
4	**521.224**	D	**809.404**	7
5	622.272	T	**694.377**	6
6	769.34	F	**593.329**	5
7	**826.362**	G	**446.261**	4
8	955.404	E	**389.239**	3
9	1068.488	L	260.197	2
10		K	147.113	1

Precursor 927.552Da MS/MS results (protein ID 1821 - Fatty acid-binding protein, epidermal)

Mascot Search Results

Match to: **FABP5_HUMAN** Score: **26**

Fatty acid-binding protein, epidermal - Homo sapiens (Human)

Matched peptides shown in **Bold Red**

1 MATVQQLEGR WRLVDSKGFD EYMK**ELGVGI ALR**KMGAMAK PDCIITCDGK

51 NLTIKTESTL KTTQFSCTLG EKFEETTADG RKTQTVCNFT DGALVQHQEW

101 DGKESTITRK LKDGKLVVEC VMNNVTCTRI YEKVE

Start - End	Observed	Mr(expt)	Mr(calc)	ppm	Miss	Sequence
**25 - 33**	**927.552**	**926.545**	**926.555**	**-11**	**0**	**K.ELGVGIALR.K** **(Ions score 26)**

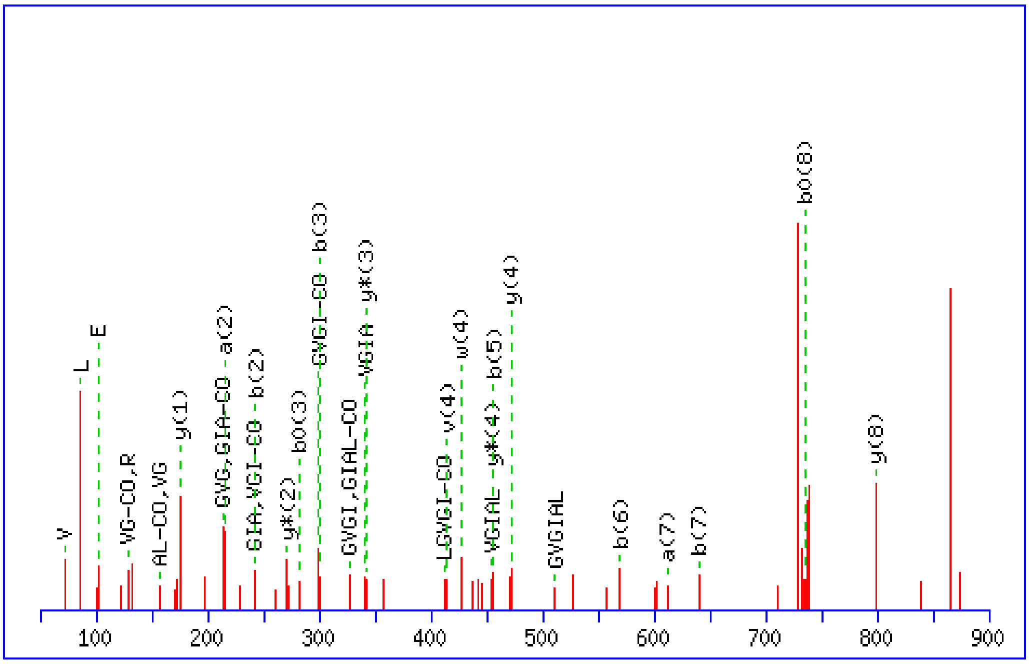

#	b	Seq.	y	#
1	130.05	E		9
2	243.134	L	798.52	8
3	300.155	G	685.436	7
4	399.224	V	628.414	6
5	456.245	G	529.346	5
6	569.329	I	472.324	4
7	640.366	A	359.24	3
8	753.451	L	288.203	2
9		R	175.119	1

Precursor 1388.841Da MS/MS results (protein ID 2120 - Poly(rC)-binding protein 1)

Mascot Search Results

Match to: **PCBP1_HUMAN** Score: **36**

Poly(rC)-binding protein 1 - Homo sapiens (Human)

1 MDAGVTESGL NVTLTIRLLM HGKEVGSIIG KKGESVKRIR EESGARINIS

51 EGNCPERIIT LTGPTNAIFK AFAMIIDKLE EDINSSMTNS TAASRPPVTL

101 RLVVPATQCG SLIGKGGCKI KEIRESTGAQ VQVAGDMLPN STERAITIAG

151 VPQSVTECVK QICLVMLETL SQSPQGRVMT IPYQPMPASS PVICAGGQDR

201 CSDAAGYPHA THDLEGPPLD AYSIQGQHTI SPLDLAKLNQ VARQQSHFAM

251 MHGGTGFAGI DSSSPEVKGY WASLDASTQT THELTIPNNL IGCIIGRQGA

301 NINEIRQMSG AQIKIANPVE GSSGRQVTIT GSAASISLAQ YLINARLSSE

351 KGMGCS

Start - End	Observed	Mr(expt)	Mr(calc)	ppm	Miss	Sequence
58 - 70	1388.841	1387.833	1387.807	19	0	R.IITLTGPTNAIFK.A

(Ions score 36)

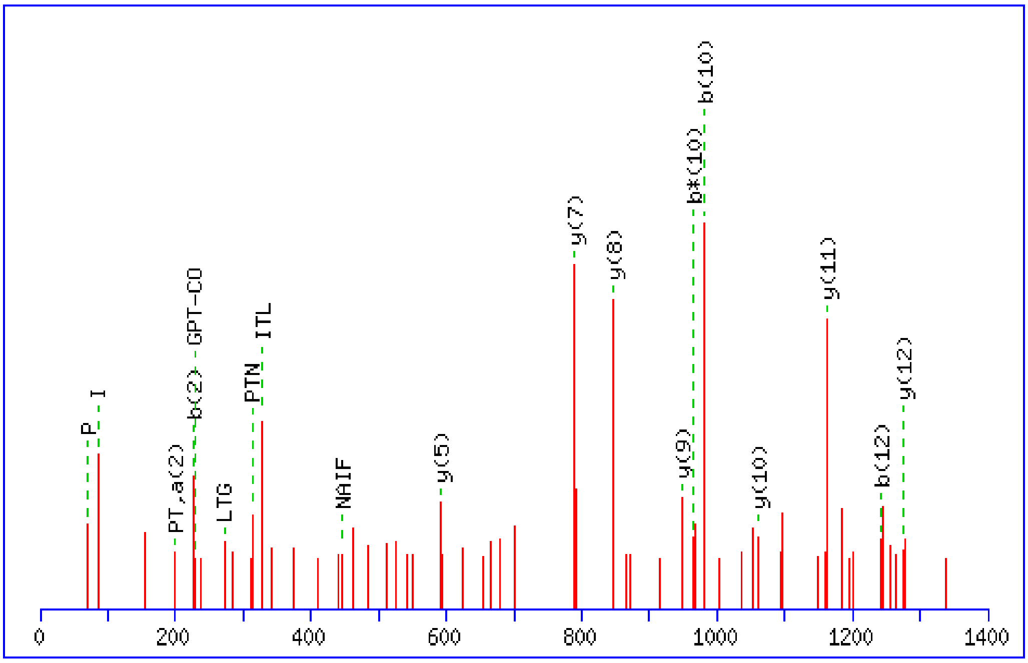

#	b	Seq.	y	#
1	114.091	I		13
2	227.175	I	1275.731	12
3	328.223	T	1162.647	11
4	441.307	L	1061.599	10
5	542.355	T	948.515	9
6	599.376	G	847.467	8
7	696.429	P	790.446	7
8	797.477	T	693.393	6
9	911.52	N	592.345	5
10	982.557	A	478.302	4
11	1095.641	I	407.265	3
12	1242.709	F	294.181	2
13		K	147.113	1

Precursor 914.507Da MS/MS results (protein ID 2120 - Heterogeneous nuclear ribonucleoprotein D0)

Mascot Search Results

Match to: **HNRPD_HUMAN** Score: **44** Expect:

0.77

Heterogeneous nuclear ribonucleoprotein D0 - Homo sapiens (Human)

Matched peptides shown in **Bold Red**

1 MEVPPRLSHV PPPLFPSAPA TLASRSLSHW RPRPPRQLAP LLPSLAPSSA

51 RQGARRAQRH VTAQQPSRLA GGAAIKGGRR RRPDLFRRHF

KSSSIQRSAA

101 AAAATRTARQ HPPADSSVTM EDMNEYSNIE EFAEGSKINA

SKNQQDDGKM

151 FIGGLSWDTS KKDLTEYLSR FGEVVDCTIK TDPVTGRSR**G FGFVLFK**DAA

201 SVDKVLELKE HKLDGKLIDP KRAKALKGKE PPKKVFVGGL SPDTSEEQIK

251 EYFGAFGEIE NIELPMDTKT NERRGFCFIT YTDEEPVKKL LESRYHQIGS

301 GKCEIKVAQP KEVYRQQQQQ QKGGRGAAAG GRGGTRGRGR

GQGQNWNQGF

351 NNYYDQGYGN YNSAYGGDQN YSGYGGYDYT GYNYGNYGYG

QGYADYSGQQ

401 STYGKASRGG GNHQNNYQPY

Start - End	Observed	Mr(expt)	Mr(calc)	ppm	Miss	Sequence
**190 - 197**	**914.507**	**913.500**	**913.506**	**-7**	**0**	**R.GFGFVLFK.D (Ions score 36)**

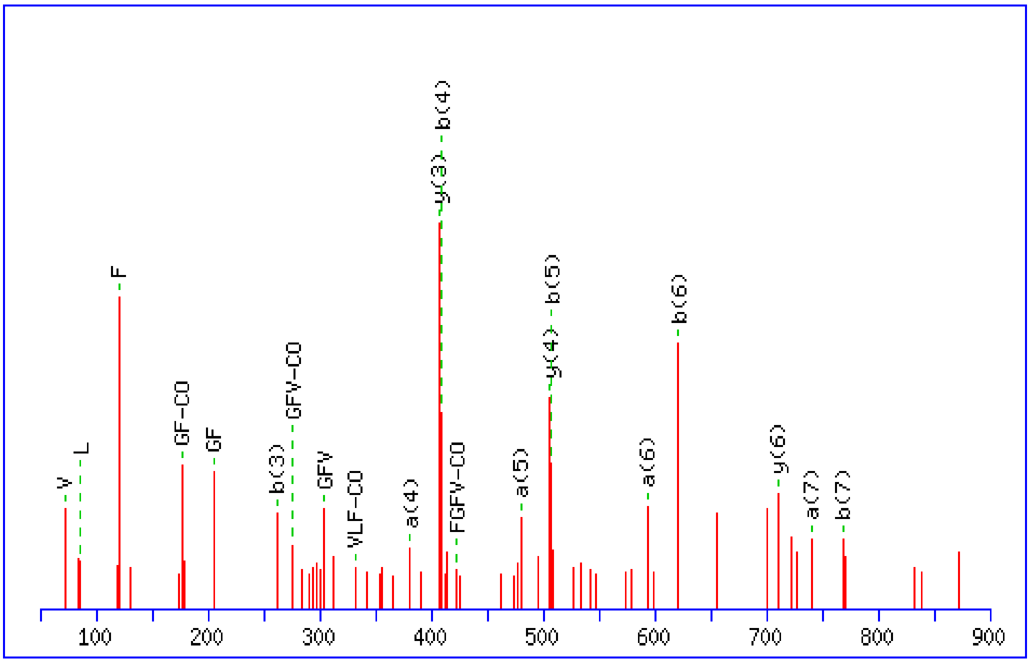

#	b	Seq.	y	#
1	58.029	G		8
2	205.097	F	857.492	7
3	262.119	G	710.424	6
4	409.187	F	653.402	5
5	508.255	V	506.334	4
6	621.34	L	407.265	3
7	768.408	F	294.181	2
8		K	147.113	1
