# Supplementary material for: Identification of target antigens of anti-endothelial cell and anti-vascular smooth muscle cell antibodies in patients with giant cell arteritis: a proteomic approach
Source: Arthritis Res Ther. 2011 Jun 28;13(3):R107. doi: 10.1186/ar3388 (PMC3218922; doi:10.1186/ar3388)
Supplement: Additional file 7 — Supplemental Table S3. Antigens specifically recognised by IgG of two-thirds of the pools of sera from giant cell arteritis patients. [file ar3388-S7.DOC]

**Supplemental table S3. Antigens specifically recognized by IgG of two-third pools of sera from giant cell arteritis patients.** Protein extract is from human umbilical vein endothelial cell.

| Spot ID | Protein | GCA 1 | GCA 2 | GCA 3 |
| --- | --- | --- | --- | --- |
| 228 | Vinculin | + |  | + |
| 461 | Lamin A/C |  | + | + |
|  | Semaphorin-4D precursor |  | + | + |
| 476 | Ezrin |  | + | + |
|  | Moesin |  | + | + |
|  | Lamin A/C |  | + | + |
|  | Radixin |  | + | + |
|  | Semaphorin-4D precursor |  | + | + |
| 557 | Far upstream element-binding protein 1 |  | + | + |
| 631 | Lamin A/C | + | + |  |
| 646 | Lamin A/C | + | + |  |
| 680 | No protein identified | + | + | + |
| 681 | No protein identified | + | + |  |
| 683 | No protein identified | + | + | + |
| 703 | No protein identified | + | + | + |
| 768 | No protein identified | + |  | + |
| 784 | Dihydrolipoyl dehydrogenase, mitochondrial precursor | + | + | + |
| 789 | Inosine-5'-monophosphate dehydrogenase 2 | + | + | + |
| 853 | No protein identified | + |  | + |
| 908 | Alpha-enolase | + |  | + |
| 950 | Tripeptidyl-peptidase 1 precursor | + |  | + |
| 1017 | Fumarate hydratase, mitochondrial precursor | + |  | + |
| 1085 | Heterogeneous nuclear ribonucleoprotein D0 | + | + |  |
| 1214 | PDZ and LIM domain protein 1 | + |  | + |
| 1249 | 60S acidic ribosomal protein P0 | + |  | + |
| 1352 | Voltage-dependent anion-selective channel protein 2 | + | + | + |
| 1359 | Annexin A5 |  | + | + |
| 1376 | No protein identified | + |  | + |
| 1440 | Heat shock protein beta-1 |  | + | + |
|  | NADH dehydrogenase [ubiquinone] iron-sulfur protein 3, mitochondrial precursor |  | + | + |
| 1614 | Protein DJ-1 |  | + | + |
| 1632 | No protein identified |  | + | + |
| 1734 | Peptidyl-prolyl cis-trans isomerase A | + |  | + |
| 1817 | Thioredoxin-dependent peroxide reductase, mitochondrial precursor | + | + |  |
| 1821 | Fatty acid-binding protein, epidermal | + | + |  |
| 2120 | Elongation factor Tu, mitochondrial precursor |  | + | + |
|  | Poly(rC)-binding protein 1 |  | + | + |
|  | Heterogeneous nuclear ribonucleoprotein D0 |  | + | + |

GCA: Giant cell arteritis; ID: Identity; PDZ and LIM domain protein 1: Post-synaptic density 95 (PSD95), Drosophila disc large tumor suppressor (DlgA), zonula occludens-1 protein (zo-1) and Lin-11, Isl-1 and Mec-3 domain protein 1
